# Adjusting the Composition of Novel Earth‐Abundant Transition Metal‐Based Oxide Nanoparticles for Electrocatalytic Oxygen Evolution Reaction

**DOI:** 10.1002/cssc.70880

**Published:** 2026-07-11

**Authors:** Julia P. Wölfel, Roland Marschall

**Affiliations:** ^1^ Physical Chemistry III University of Bayreuth Bayreuth Germany

**Keywords:** electrocatalysis, high‐entropy, oxygen evolution, spinel, transition metals

## Abstract

To produce green hydrogen through water electrolysis, highly efficient catalysts that use inexpensive, earth‐abundant materials are required. In search of novel and inexpensive electrocatalysts, we investigate the adjustment of the composition of transition metal‐based catalysts for the alkaline oxygen evolution reaction (OER) and evaluate the effects of both redox‐active and ‐inactive elements. Based on the known synthesis of AFe_2_O_4_‐type spinels (A = transition metal cation), we adjusted the composition of the A and B positions (B = Fe position), achieving a significant improvement in catalytic activity. Additionally, we are investigating the high‐entropy effect, which is expected to enhance the activity and stability of electrocatalysts. Introducing cobalt and manganese and combining these elements with nickel and zinc formed a NiO/(NiZn)(MnCoFe)_2_O_4_ composite that reduced the overpotential for OER to 360 mV. However, increasing the number of A‐cations in the spinel beyond six to reach a high‐entropy spinel oxide had no positive effect on the overpotential. In terms of composition, the amount of nickel, and thus NiO, could be reduced to 5%, maintaining low overpotentials. The same applies to the proportion of cobalt, which does not scale linearly with activity.

## Graphical abstract

This work investigates how the ability of transition‐metal spinel oxides to perform the oxygen evolution reaction (OER) depends on their composition, ranging from compositionally complex oxides to high‐entropy oxides. The materials are composed of different transition metals in varying ratios and quantities to study the effects of increasing the number of elements. During this investigation, a NiO/spinel composite is found that can greatly enhance OER activity.

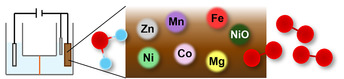

## 1 Introduction

The production of green hydrogen through water electrolysis is a highly relevant topic in the modern age facing climate change due to the emission of greenhouse gases [1, 5]. With a chemical energy density of 140 MJ kg^−1,^ hydrogen combines the possibility of a zero‐emission energy cycle with one of the highest energy storage capacities, making it a great alternative to fossil fuels [6, 8]. Despite numerous publications focusing on exploring and optimizing electrode materials, the search for a precious metal‐free, sustainable, and highly active catalysts for alkaline oxygen and hydrogen evolution reaction (HER and OER) is still ongoing [9, 10]. Especially the oxygen evolution reaction (OER) can be considered the bottleneck of overall water splitting (OWS), since four electrons need to be transferred [11, 12].

The most observed reaction mechanism is the adsorbed evolution mechanism (AEM), which is a four‐step reaction, including four intermediates with different adsorption energies. In a first step, OH^‒^ is adsorbed on a metal active site, then followed by deprotonation forming an M─O species. With another OH^‒^ an O─O bond in the form of a surface M─OOH is formed, which is converted into O_2_ by a final protonation [13, 15]. Next to AEM, the second relevant mechanism is the lattice oxygen mechanism (LOM), which can explain significantly lower overpotentials and structural changes in catalyst materials during electrocatalysis [16, 17]. A lattice oxygen takes part in the O_2_ formation, leaving a vacancy in the crystal structure, which is then filled by the oxygen of a water/hydroxide molecule [15]. The amount of lattice oxygen involved in the OER is known to be significant for the activity achieved, which in turn depends on the composition and crystal structure of the catalyst [18, 19]. Adjusting the electronic structure via compositional changes is a known strategy to promote the LOM contribution to a catalytic reaction [15, 20, 21]. Simultaneously, a more amorphous catalyst is likely to allow more continuous reconstruction of its surface due to the already existing long‐range disorder and defects [22, 23]. A general understanding of the underlying OER mechanism on the catalyst's surface is crucial to adapt its properties to reaction conditions.

In the past years, transition‐metal‐based materials became more popular in electrocatalyst design due to their earth abundance and lower costs compared to precious metals like platinum or iridium [24]. Redox‐active elements, especially nickel (Ni), cobalt (Co), or iron (Fe), are commonly known for their broad application possibilities in electrocatalytic reactions [6, 12, 25]. Bi‐ and trimetallic systems are widely investigated as OER and HER catalysts; activity is thereby promoted by the synergies of the transition metals [6, 26]. The downside of utilizing a large amount of Co or Ni is still their toxicity and the harvesting/production in certain countries [27]. Replacing or at least reducing these elements in modern catalytic materials is a challenge to fulfill while keeping materials stable and highly active, which can be achieved by combining these active elements with redox‐inactive elements like Zn, which simultaneously can promote LOM contribution and keep activity stable at high current densities [28].

Next to the compositional adjustment in a catalyst material, the high entropy effect also comes into play once a certain number of elements is reached. It is expected to have a significant influence on catalytic properties due to the stabilization of the material in a higher energetic state and the synergies between multiple elements creating various surface adsorption energies [29, 30]. To reach a single‐phase material ( $\text{Δ}G_{\mathrm{mix}\;}<0$, Formula (1) stabilized by high entropy, the entropy contribution must overcome the enthalpy part ($\text{Δ}S_{\mathrm{mix}}>\text{Δ}H_{\mathrm{mix}}$) [31, 32].

(1) $$\text{Δ}G_{\mathrm{mix}\;}=\text{Δ}H_{\mathrm{mix}}-T\text{Δ}S_{\mathrm{mix}}$$

Although entropy has different contributions like configurational, vibrational, electron random, or magnetic dipole entropy, most of them can be neglected apart from configurational entropy [33, 34]. Based on its calculation (Formula (2) on the cation site, at least five elements in equimolar amounts must be combined in one (sub)lattice to reach high entropy stabilization [35, 36].

(2) $$\text{Δ}S_{\text{config}}=-R\left[x{\left(\sum_{i=1}^{n}x_{i}\ln\;x_{i}\right)}_{\text{cation}-\mathrm{site}}\right]$$

Multiple spinel‐type high‐entropy materials are applied in electrocatalysis with varying synthesis routes. Commonly, all these methods require extreme conditions such as high temperature or pressure to form phase‐pure high‐entropy oxide (HEO) materials [19]. However, the application of HEOs in OER catalysis shows promising results. The combination of transition metals in materials like (CrMnFeCoNi)_3_O_4_, (CoNiMnZn)Fe_2_O_4_, and (FeNiCoCrCu)_3_O_4_ with overpotentials of 390 mV, 276 mV, and 270 mV emphasizes the potential in HEO materials [37, 39]. In more recent studies, the influence of high entropy and elemental composition on the underlying OER mechanism is also studied, for example in (CoFeNiMnW)_3_O_4_, where a low overpotential of 256 mV was reached due to LOM activation [40].

Apart from composition, activity will also depend on the chosen synthesis route, as well as the electrode material used during OER. To exclude participation of the electrode itself in the water splitting, as Ni foam, for example, can also form active surface species like NiOOH; here Carbon Paper (CP) is used as electrode [41].

Recently, we demonstrated that synthesis of HEO spinels (AFe_2_O_4_) and tailoring of their A position was possible via a low‐temperature, microwave‐assisted synthesis route. We also showed that including Co in the Fe(B) position significantly increased OER activity compared to adjusting the A position [42]. Building on these results, this work focuses on tailoring the B‐ position composition and includes the transition metal manganese (Mn) to investigate its influence on the OER overpotential. Additionally, we adjust the A position with both redox‐active and inactive elements. This allows for a more complex crystal structure with various possible distributions of elements across the A and B positions, as all three base elements are stable in oxidation states 2+ and 3+. This significantly influences the resulting activity towards OER because there is a direct connection between composition and overpotential. Introducing other elements affects material properties, such as active surface area and charge transfer. These properties directly impact electrocatalytic activity. These effects can be reversed or reinforced by synergies between the additional elements.

## 2 Experimental Section

### 2.1 Spinel Synthesis

Spinel nanoparticles were synthesized according to our previously reported nonaqueous microwave‐assisted synthesis [42]. In the case of an (A)(B)_2_O_4_ structured spinel, 0.5 mmol of A(acac)_2_ precursor (A = Ni, Zn, Mg) and 1.0 mmol B(acac)_3_ (B = Mn, Co, Fe) precursor in total were used. In the case of (M)_3_O_4_ (M = Ni, Zn, Mg, Mn, Co, Fe) structured spinels, equimolar amounts of M(acac)_2/3_ precursor were used to reach 1.5 mmol metal precursor in total. The precursors were dissolved in 15 mL of 1‐Phenylethanol and stirred overnight. The solution was transferred to a borosilicate microwave vessel and placed in the microwave for 30 minutes at 225°C (Anton Paar Monowave 400, heating with constant power of 30 W until 200°C, then with 35 W until 225°C, stirring speed 800 rpm) and cooled with compressed air. The product was precipitated with n‐pentane and washed once with acetone, twice with acetone/water (3/1), and once with diethyl ether. The final product was then dried at 80°C overnight in air.

### 2.2 Characterization

The Ag‐XRD measurements were performed in a STOE STADI P Mythen2 4 K diffractometer (Ge (111) monochromator and four DectrisMYTHEN2 R 1 K strip detectors) using Kα irradiation in transmission geometry [43]. The powders were measured in Hildenberg capillaries (outer diameter 0.5 mm). To analyze the XRD patterns, the software X’Pert HighScore Plus was used. Reference patterns were obtained by simulation with VESTA. Crystallite sizes were calculated with the Scherrer equation. Raman measurements were performed with a Horiba Yvon Raman microscope and an 11.5 W He‐Ne laser (λ = 633 nm); the intensity was reduced to prevent heating. For DRIFT (Diffuse Reflectance Infrared Fourier Transform) spectra, a Bruker Alpha II spectrometer was used. Nitrogen physisorption measurements were conducted with a Quadrasorb Evo and a Nova 800 device from Anton Paar QuantaTec at 77 K. Samples were degassed for 12 h at 120°C before the measurements. The surface area was evaluated with the software ASiQwin using the Brunauer–Emmet–Teller (BET) model. Scanning electron microscopy (SEM) images were taken with a Zeiss Leo 1530 device at an acceleration speed of 3 kV, with Pt sputtering (Cressington Sputter Coater 208 HR). Energy‐dispersive X‐ray spectroscopy (EDX) was conducted on the same instrument at an acceleration voltage of 20 kV with an ultra‐dry EDX detector by Thermo Fisher Scientific NS7. X‐ray photon spectroscopy (XPS) was conducted on a Physical Electronics PHI VersaProbe III Scanning XPS Microprobe instrument. Al K_α_ irradiation, with 15 kV beam voltage, 50 W power, and 200 µm beam diameter, was used. For the survey spectra, a 0.4 eV step size, 50 ms time per step, and 224 eV pass energy were applied; and for the high‐resolution spectra, 0.1 eV step size, 50 ms time per step, and 26 eV pass energy were applied. The samples were continuously flooded with Ar^+^ and electrons at low energy. Data analysis was performed with Casa XPS, Shirley Background, and a 30% Gaussian‐Lorentzian profile function (GL30) was used. The signals were corrected to C 1s 248.8 eV.

### 2.3 Electrochemical Measurements

The electrocatalytic measurements were performed with a Gamry Reference 3000. A two‐compartment glass cell separated by a Selemion membrane (AGC group) was used, schematically shown in Figure S1 (Supporting Information). All measurements were carried out in 1 M KOH, purged with Argon. A three‐electrode setup was used with a Pt net as counter electrode, a reversible hydrogen electrode (RHE, Hydroflex gaskatel) as reference electrode, and a CP electrode (Freudenberg H2315‐C2) as working electrode. For the preparation of the CP electrode, a dispersion of 10 mg of catalyst, 20 µL of 5 wt% Nafion, and 300 µL of iso‐propyl alcohol was ultrasonicated for 30 minutes and drop‐casted (2x 25 µL) on a previously prepared CP. The electrode area of the CP was restricted to 1 cm^2^ with Kapton tape. A measurement sequence of two impedance measurements (first at OCP (open circuit potential), second at 1.7 V_RHE_), a CV (cyclic voltammetry) scan between −0.6 and 2.0 V_RHE_, CV scans of different scan rates (5–200 mV/s) between 1.2 and 1.3 V_RHE_ for DLC (double layer capacitance) calculation, 25 LSV (linear sweep voltammetry) sweeps between 1.0 and 2.0 V, again impedance at 1.7 V, DLC determination, and lastly a full CV scan was carried out for every electrode. The overpotential was calculated by subtracting 1.23 V_RHE_ from the potential needed to reach 10 mA/cm^2^. The CTR (charge transfer resistance) was obtained by calculating the diameter of the measured semicircle.

All used chemicals are listed in Table S1 (Supporting Information).

## 3 Results and Discussion

This work focuses on adjusting the composition of spinels for alkaline OER. The general spinel structure, (A)(B)_2_O_4_, categorizes the elements in the +2 (tetrahedral) A position and the +3 (octahedral) B position. However, when converting to the more general formula (M)_3_O_4_, no distinction is made between the oxidation states of +2 and +3, nor the theoretical occupation of the A and B positions. When including elements that are only stable in the +2 oxidation state, such as zinc (Zn), the ratio of +2 to +3 elements must be considered to ensure charge neutrality. The composition notation in this work displays only the intended theoretical occupation of the crystallographic positions, while also representing the elemental ratio. Due to highly disordered materials, a wide range of compositions, and possible inversion of the spinel structure, it is impossible to analyze the actual crystallographic position of each element and its environment.

**FIGURE 1:**
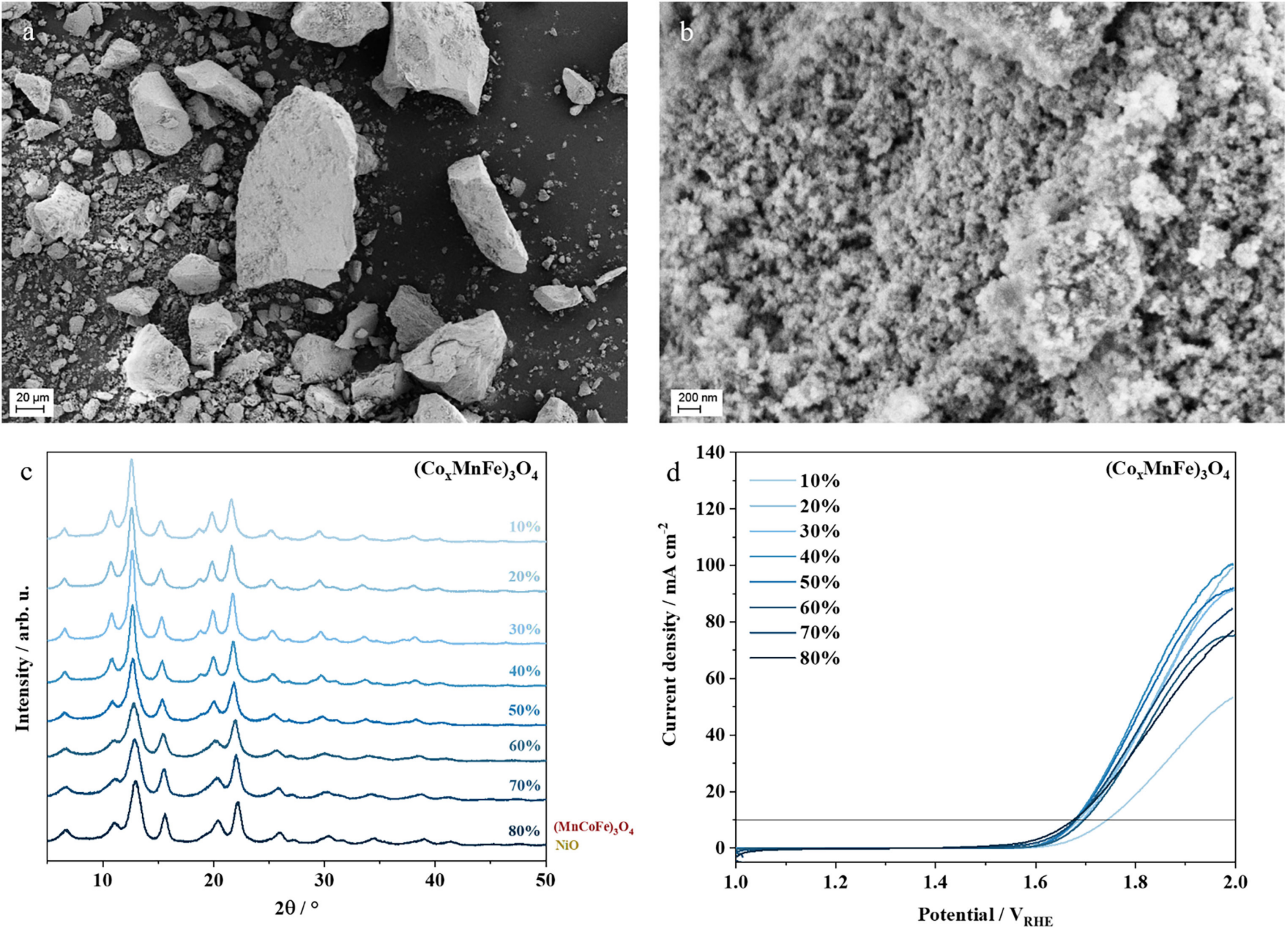
(a) and (b) SEM images of (MnCoFe)_3_O_4_ in two magnifications; (c) Ag‐XRD of varying Co‐content in the trimetallic (Co_*x*_MnFe)_3_O_4_ spinel, x% indicates the total amount of Co, remaining parts are filled with equimolar amounts of Fe and Mn; (d) corresponding LSV measurements, performed in 1 M KOH; CTR and ECSA determination, CV scans, and Tafel analysis given in Figures S4 and S5 and Table S3, Supporting Information.

The starting composition for this study is the trimetallic spinel (MnCoFe)_3_O_4_, which forms a phase‐pure spinel structure with predominantly amorphous nanoparticles with a crystallite size of approximately 6.0–12.0 nm. By assuming an equimolar distribution of all three elements over both A and B positions, its entropy, based on Formula (2), can be calculated to 1.099 R, which classifies this material as medium entropy. However, due to possible inversion and preferred oxidation states of individual elements, this calculation is not straightforward [44]. These nanoparticles accumulate in larger agglomerates of varying sizes in the nm to µm range (Figure 1a,b). XPS measurements (Figure S2, Supporting Information) confirm the incorporation of each element into the spinel structure. Due to overlapping signals, their ratios can only be estimated, but roughly fit the intended values (Table S2, Supporting Information). O‐values are not discussed due to the remaining organic residues on the particles.

The amount of Co was then investigated between 10% and 80%, which resulted in a decrease in entropy compared to the equimolar ratios between 0.639 R (10% Co) and 0.949 R (80% Co), categorizing these catalysts as low entropy. This had no influence on the phase purity of the catalysts, as seen in Figure 1c. Raman and DRIFT characterization are given in Figure S3 (Supporting Information), verifying low crystallinity and organic residues on the particle’s surface. Although the initial incorporation of Co had previously increased the electrocatalytic activity, and Co is known for its catalytic properties, no direct correlation was found between Co amount and the overpotential achieved or the overall activity, that is, the maximum current density (Figure 1d). The lowest overpotentials are reached with 30% to 40% Co, in the range of nearly equimolar ratios. Co‐active sites by themselves are therefore not the only origin of activity, which indicates the importance of a precisely tailored composition in spinel electrocatalysts.

### 3.1 Addition of Redox Active and Inactive Elements on the A Position

**FIGURE 2:**
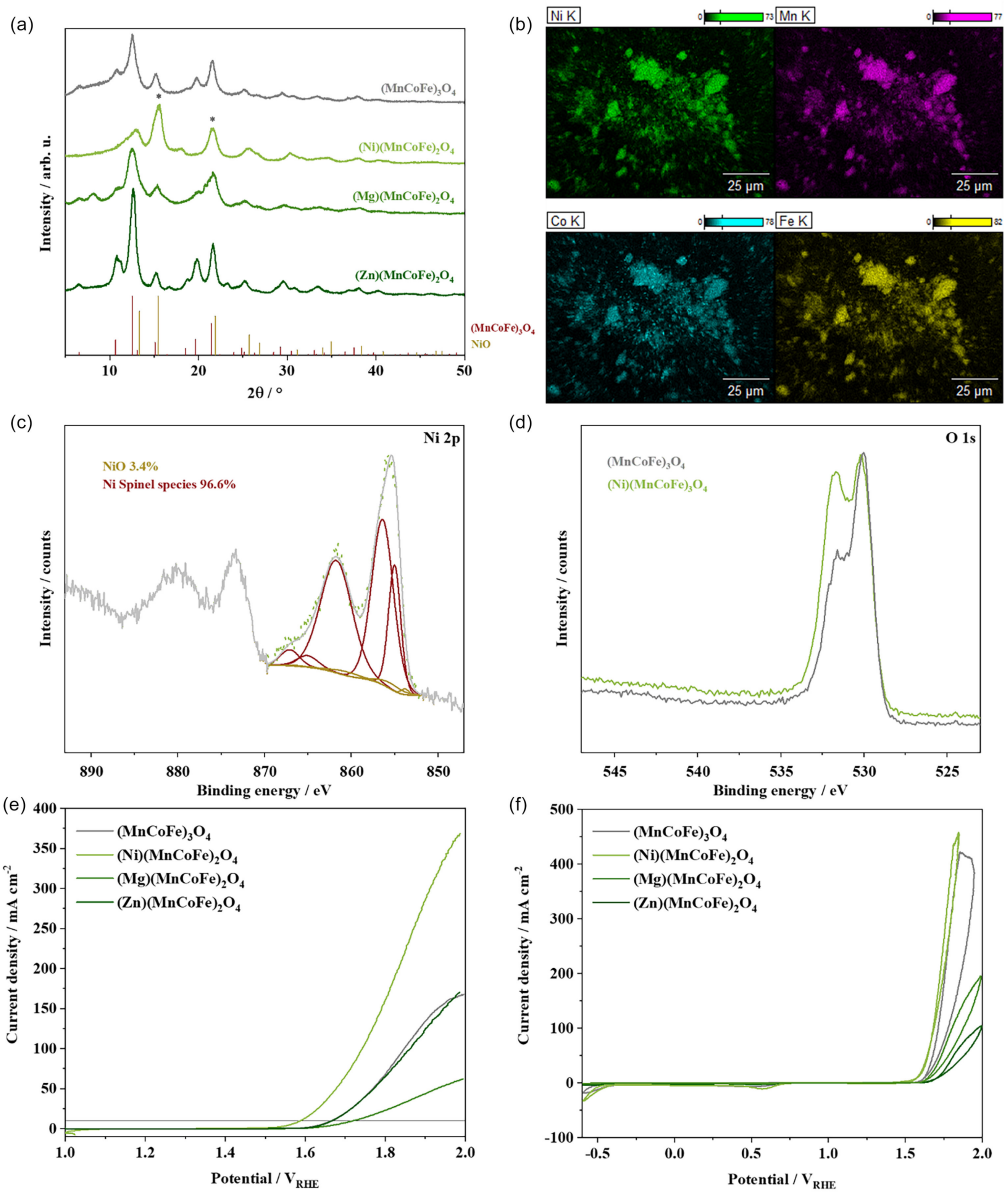
(a) Ag‐XRD with added redox‐active and inactive elements; (b) EDX mapping of (Ni)(MnCoFe)_2_O_4_; (c) XPS fit of high‐resolution Ni 2p spectra; (d) XPS high‐resolution O 1s spectra of (MnCoFe)_3_O_4_ and (Ni)(MnCoFe)_2_O_4_; (e) LSV measurements and (f) CV measurements performed in 1 M KOH.

Adding an element that is only stable in an oxidation state of +2 presumably fixes the existing redox‐active elements Co, Mn, and Fe in an oxidation state of +3, which increases the entropy to 1.369 R and classifies these materials as medium entropy, if a perfect solid solution is present. This can, again due to inversion, not be ensured. Adding both the redox‐inactive elements Zn (ionic radius: 60 pm) and magnesium (Mg, ionic radius: 72 pm) and the redox‐active transition metal Ni (ionic radius: 55 pm) can give insights into the resulting synergies between these elements and their influence on overpotentials [45]. Zn and Mg are incorporated into the spinel without the formation of any side phases. When Ni is added, an NiO side‐phase forms, as the intensity distribution in the XRD no longer corresponds to the typical spinel pattern (Figure 2a). The crystallite sizes (given in Table S4, Supporting Information) change when different elements are added due to their ionic radii, resulting in slightly different structural properties such as lower crystallinity. EDX mapping (Figure 2b) of (Ni)(MnCoFe)_2_O_4_ shows a homogeneous distribution of all elements across the particle surface, with 34% Ni, 22% Mn, 22% Co, and 22% Fe. The NiO phase, therefore, forms invisible clusters and is distributed over the entire surface in the form of a composite‐like structure. SEM images show agglomerates of different sizes; a side phase is not detectable. HR‐TEM images confirm agglomerates of nanocrystalline particles that are randomly orientated with a lattice plane distance of ∼0.24 nm (Figure S6, Supporting Information). Since it is clearly visible in the XRD, an NiO phase should be detectable in XPS. The XPS survey scan confirms the presence of all elements; again, ratios are only roughly estimated due to overlapping signals (Figure S7 and Table S5, Supporting Information). In the high‐resolution spectra of the Ni 2p3/2 signal (Figure 2c), about 3.4% of NiO can be detected, with most of the Ni species (96.6%) being attributed to the spinel [46, 47]. This suggests that the majority of Ni is incorporated into the spinel, which forms an almost completely amorphous material and is therefore virtually invisible in the XRD. At the same time, NiO is more crystalline and, although present in only small amounts, detectable in XRD. When comparing the high‐resolution O 1s spectra (Figure 2d) of (MnCoFe)_3_O_4_ and (Ni)(MnCoFe)_2_O_4_, no major shifts or additional oxygen signals can be detected that could indicate larger amounts of an additional secondary phase. The NiO O 1s signals would be expected at lower binding energies (∼529.3 ± 0.04 eV) [46, 48]. The O 1s signal is not analyzed further due to organic residues and the multitude of possible adsorbed oxygen species on the particle surface. The bigger signal at ∼532.0 eV is therefore attributed to organic species on the material's surface [49]. The NiO formation could be explained by a faster reaction mechanism in the microwave, caused by stronger absorption of microwave radiation by the Ni precursor.

The highly amorphous structure of the catalysts is again evident from the low intensity of the Raman spectra (Figure S8, Supporting Information), with no side phases visible. DRIFT spectra confirm organic residues from the synthesis on the particle surface. When applying the modified spinels in electrocatalysis (Figure 2e, Table 1), the activity and overpotential of the Zn‐containing material are comparable to the (MnCoFe) combination at 437 mV, decrease to 491 mV with Mg, and drastically improve with the formation of the NiO/spinel composite with the lowest value of 357 mV.

**TABLE 1:** Summary of electrocatalytic results, overpotential, CTR, ECSA (measured in 1 M KOH), and BET surface area for comparison.

Composition	Overpotential@10 mA, mV	CTR, Ω	ECSA, cm^2^ a	BET, m^2^ g^−1^
(MnCoFe)_3_O_4_	435	5.8	3.38	144
(Ni)(MnCoFe)_2_O_4_	357	2.3	12.33	164
(Mg)(MnCoFe)_2_O_4_	491	11.7	1.42	175
(Zn)(MnCoFe)_2_O_4_	437	4.9	7.00	167

a Calculated from double layer capacitance, fit given in Figure S10, Supporting Information.

The second important parameter for evaluation activity is the CTR, which is directly related to the trend in overpotential. Accordingly, the NiO/spinel composite has the lowest CTR of 2.3 Ω, as well as the lowest Tafel slope of 12.0 mV/dec (Figure S9, Supporting Information). The stability of the catalysts over a broad potential range is shown in CV measurements (Figure 2f), which also shows a small reduction feature at 0.5 V_RHE_ attributed to NiO, as well as some activity towards HER at high overpotentials. The oxidation of Ni likely takes place above 1.5 V_RHE_, as there is no additional oxidation feature present in the CV. This oxidation in combination with several reduced species on the catalyst's surface explains the significantly higher activity and lower overpotential in CV compared to LSV. The overpotentials given are therefore derived from LSV to ensure comparable values from a stable system.

Next to CTR, the BET surface area and especially electrocatalytic active surface area (ECSA) are crucial for OER activity, as it represents the amount of active surface that takes part in the electrocatalytic reaction. All catalysts are nanoparticles and therefore have a high BET surface area (Table 1). The ECSA (after LSV) shows significant differences that correlate with the measured overpotentials, as the highest ECSA of 12.33 cm^2^ was found for (Ni)(MnCoFe)_2_O_4_.

**FIGURE 3:**
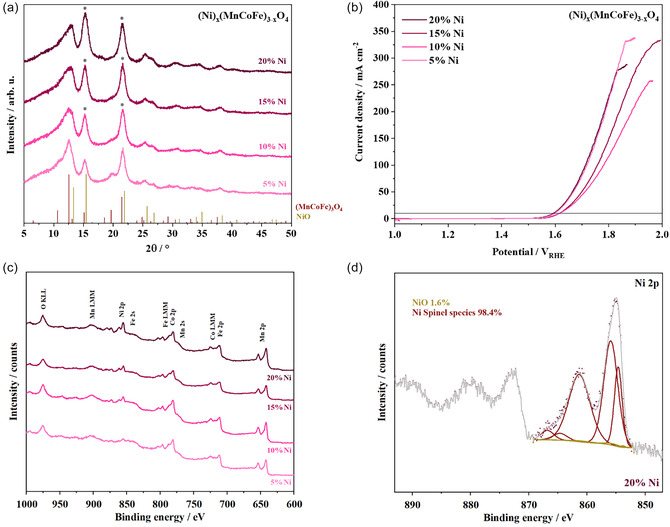
(a) Ag‐XRD of spinels with varying Ni‐content, (b) corresponding LSV measurements, performed in 1 M KOH and (c) Enlargement of XPS survey scan of (Ni)_*x*_(MnCoFe)_3‐x_O_4_, full spectra given in Figure S11, Supporting Information (d) exemplary Ni 2p high‐resolution spectra with NiO fitting, spectra for 15%, 10%, and 5% Ni given in Figure S13, Supporting Information; further characterization given in Figure S14, Supporting Information.

The incorporation of Ni into the spinel structure, as well as the combination with small amounts of NiO, led to a drastic increase in activity. Therefore, the amount of Ni in the catalysts was further varied. Below 5% Ni, an NiO side phase is no longer detectable in the XRD (Figure 3a). The BET surface area and ECSA do not vary significantly with the amount of Ni (Table 2). XPS survey scans confirm a changing amount of Ni in the catalysts, matching the intended decrease of total Ni (Figure 3c, Figure S11, Table S6, Supporting Information). The Ni 2p high‐resolution spectra show a constant amount of 96%–98% Ni incorporated in the spinel (Figure 3d), and therefore also a constant amount of 2%–4% NiO, slight variations occur due to the quality of the high‐resolution spectra itself. The overpotentials and current densities achieved are similar for all amounts; small differences may be caused by electrode preparation and material detachment during measurement due to intense O_2_ bubbling (Figure 3b). Since the electrocatalytic activity is not affected by the total amount of Ni incorporated into the spinel, the introduction of some Ni into the spinel is sufficient for the increased activity towards OER, as well as a small percentage of NiO, which are the crucial parameters for increasing OER activity, as the changing spinel:Ni ratio is not significantly influencing the overpotential. The expected importance of an in situ formation of the NiO/spinel composite is tested by mixing 3wt% of NiO with 97wt% (MnCoFe)_3_O_4_. Since there is no change in the overpotential or overall current density with and without NiO, this indicates the importance of the in situ formation of NiO and its combination with Ni in the spinel. This might be attributed to a better charge transfer between the active site and the electrode and a broader distribution of NiO over the surface (Figure S12, Supporting Information). The in‐situ formation of NiO could have several advantages over physical mixing with NiO. It could ensure better contact between the spinel and the NiO, which could be favorable for charge transfer due to growth on top of/between the spinel nanoparticles. Moreover, in‐situ formation might result in different NiO particle sizes in contrast to those obtained by post‐synthetic addition of NiO. Consequently, one could expect a more homogeneous distribution over the spinel surface due to the in‐situ formation during synthesis. It is important to note that in the in situ‐formed heterojunctions, there is additional Ni in the spinel structure, which is crucial for OER. Such incorporated Ni is not present after the post‐synthetic addition of NiO.

**TABLE 2:** Correlation of total Ni‐amount, NiO‐amount and electrocatalytic properties, overpotential, CTR (Figure S15, Supporting Information), ECSA, performed in 1 M KOH, and BET surface area for comparison.

Total Ni‐amount, %	NiO‐amount, %^a^	Overpotential@10 mA, mV	CTR, Ω	ECSA, cm^2^ b	BET, m^2^ g^−1^
20	1.6	363	2.7	13.91	191
15	2.8	385	2.8	8.57	211
10	4.2	385	3.6	9.31	205
5	1.3	368	2.3	10.74	203

a Calculated from XPS.

b Calculated from double layer capacitance, fit given in Figure S16, Supporting Information.

### 3.2 Combining A‐Cations in Medium Entropy Regime

**FIGURE 4:**
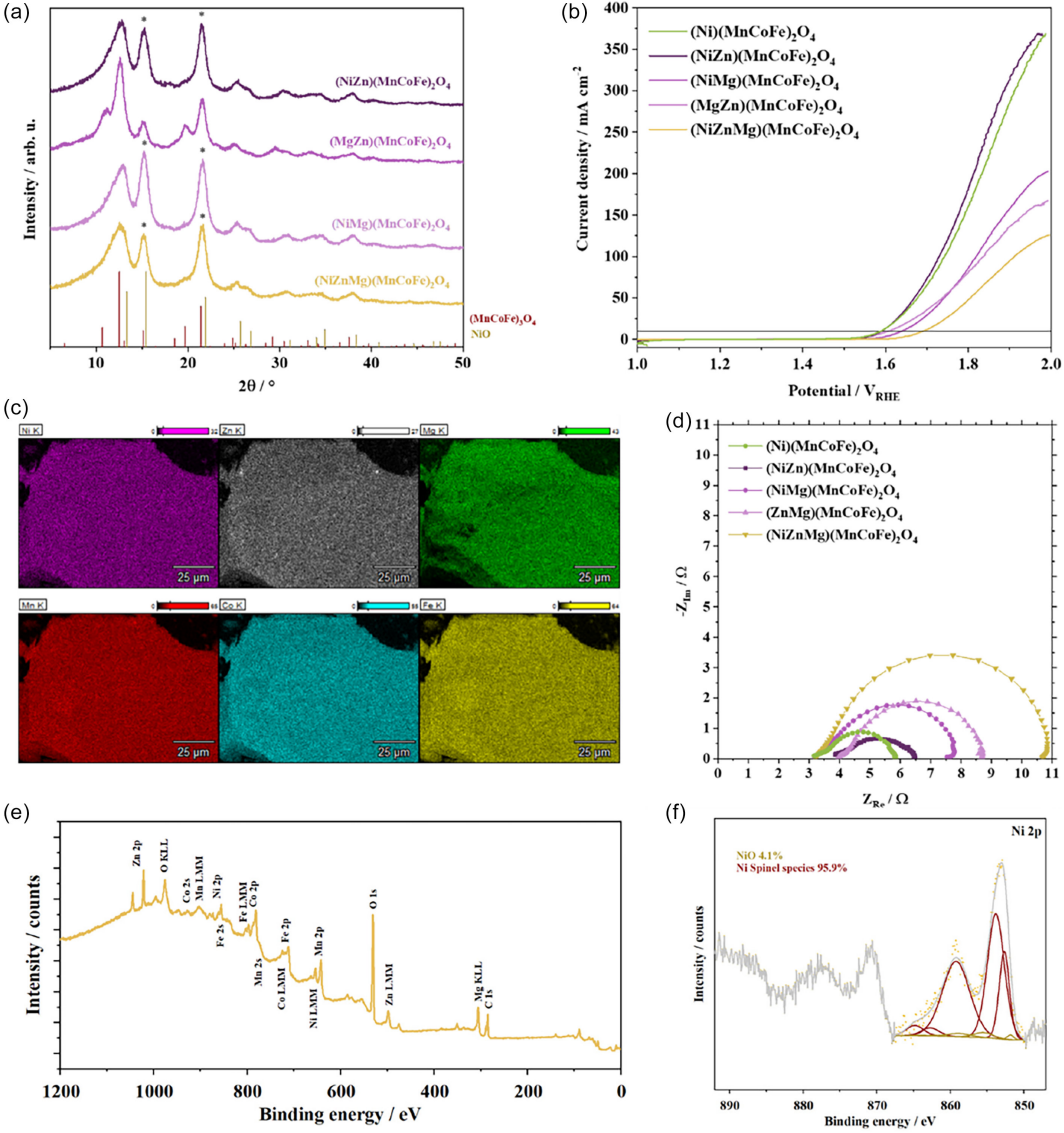
(a) Ag‐XRD of spinels combining Ni, Zn, and Mg on the A position; (b) corresponding LSV measurements, performed in 1 M KOH; CV scans and Tafel analysis given in Figure S20, Supporting Information (c) EDX Mapping of (NiZnMg)(MnCoFe)_2_O_4_; (d) EIS measurements of medium entropy catalysts; (e) XPS Survey scan of (NiZnMg)(MnCoFe)_2_O_4_; (f) High‐resolution spectra of Ni 2p signal.

Apart from adding one +2 element to the spinel structure, combining these elements could be beneficial, since reducing the amount of Ni did not reduce activity. Although the calculated entropy of 1.599 R classifies these materials as high entropy, an ideal solid solution without spinel inversion cannot be expected; therefore, they are still considered medium entropy. Synergies between the A‐cations could increase stability, influence the adsorption energies of active surface sites, or alter the LOM mechanism’s contribution to OER. However, combining Ni with Mn or Zn results in an NiO side phase, meaning that some of the Ni is not incorporated into the spinel nanoparticles (Figure 4a, further characterization Figures S17–S19, Supporting Information). The combination of (MgZn) results in larger crystals, a phase pure material is possible (Table S7, Supporting Information). EDX mapping (Figure 4c) of (NiZnMg)(MnCoFe)_2_O_4_ shows a homogeneous distribution of the elements over the entire area, the ratios of 12% Mg, 9% Zn, 11% Ni, 24% Mn, 24% Fe, and 20% Co correspond to the desired amounts of the cations. In combination with an XPS survey scan, which also confirms all intended elements in approximately matching ratios (overlapping signals, Table S8, Supporting Information), six elements are successfully combined in the NiO/spinel composite (Figure 4e). The composite in a comparable ratio between NiO and spinel‐Ni is seen in the Ni 2p high‐resolution spectra in Figure 4f. As previously indicated by the catalysts with a reduced Ni amount, the material combining Ni and Zn exhibits similar activity to (Ni)(MnCoFe)_2_O_4_, indicating that Zn is excluded from the redox reaction during OER, as it has no effect in either (Zn)(MnCoFe)_2_O_4_ or (NiZn)(MnCoFe)_2_O_4_ (Figure 4b). It is more likely to affect stability due to its fixed oxidation state of 2+. Combining Zn and Mg results in lower activity, possibly due to a decreased conductivity and the absence of NiO, which might serve as active site. A similar effect is present for the addition of (NiZnMg). The CTR results are consistent with previously determined overpotentials (Figure 4d, Table 3). The ECSA roughly follows the trend of the overpotential, increasing with lower overpotentials. The BET surface area is within the expected range for nanoparticles. The ECSA of (ZnMg)(MnCoFe)_2_O_4_ is comparatively high, but since the corresponding measurements are performed in the non‐faradaic region, redox‐active species in this potential window are not necessarily part of the electrocatalytic OER, which means that a high ECSA can indicate activity and explain differences, but does not in itself guarantee low overpotentials.

**TABLE 3:** Summary of electrocatalytic results, overpotential, CTR, ECSA (measured in 1 M KOH), and BET surface area for comparison of medium entropy spinels with two to three combined 2+ cations.

Composition	Overpotential@10 mA, mV	CTR, Ω	ECSA, cm^2^ a	BET, m^2^ g^−1^
(NiZn)(MnCoFe)_2_O_4_	360	2.3	10.91	180
(NiMg)(MnCoFe)_2_O_4_	459	7.5	6.37	170
(ZnMg)(MnCoFe)_2_O_4_	376	4.4	15.08	152
(NiZnMg)(MnCoFe)_2_O_4_	461	7.3	3.10	174

a Calculated from double‐layer capacitance, fit given in Figure S21, Supporting Information.

**FIGURE 5:**
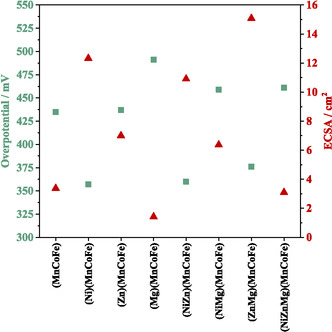
Correlation of overpotential and ECSA in low and medium entropy spinels.

The results of all medium‐ and low‐entropy materials are summarized in Figure 5. Ni addition, both alone and in combination with Zn, leads to the lowest overpotentials, a further combination with Mg is not beneficial for the OER. Furthermore, the formation of a NiO/spinel composite material results in a significantly higher ECSA. The combination of Mg and Zn has the highest ECSA and a small overpotential, but the overall current density reached is low. The combination of all three 2+ cations cannot combine a high ECSA with a low overpotential and a steep increase in current density. The active sites formed with Zn and Mg might be covered by NiO, which would result in a lower ECSA. Simultaneously, Zn and Mg reduce the conductivity of the catalyst (higher CTR) and possibly inhibit the charge transfer from NiO to the catalyst or to the electrode.

### 3.3 High Entropy Regime

Next, we investigated the high‐entropy regime and any influence on activity enhancement. High‐entropy stabilization at the A position requires combining at least five elements at this crystallographic position, which would result in a maximum entropy of 3.807 R for the whole spinel. Because multiple elements are combined in the spinel structure, inversion is likely to occur. This affects the calculation of the sublattice entropy because the occupation of the tetrahedral and octahedral vacancies changes. Thus, the A position is gradually filled with transition metals to increase entropy stabilization throughout the material. All of the catalysts in this chapter are classified as high‐entropy based on the number of metals theoretically combined at the A and B positions. Although the addition of Mg had no effect on activity, it is included to increase configurational entropy in the system and achieve high‐entropy stabilization.

**FIGURE 6:**
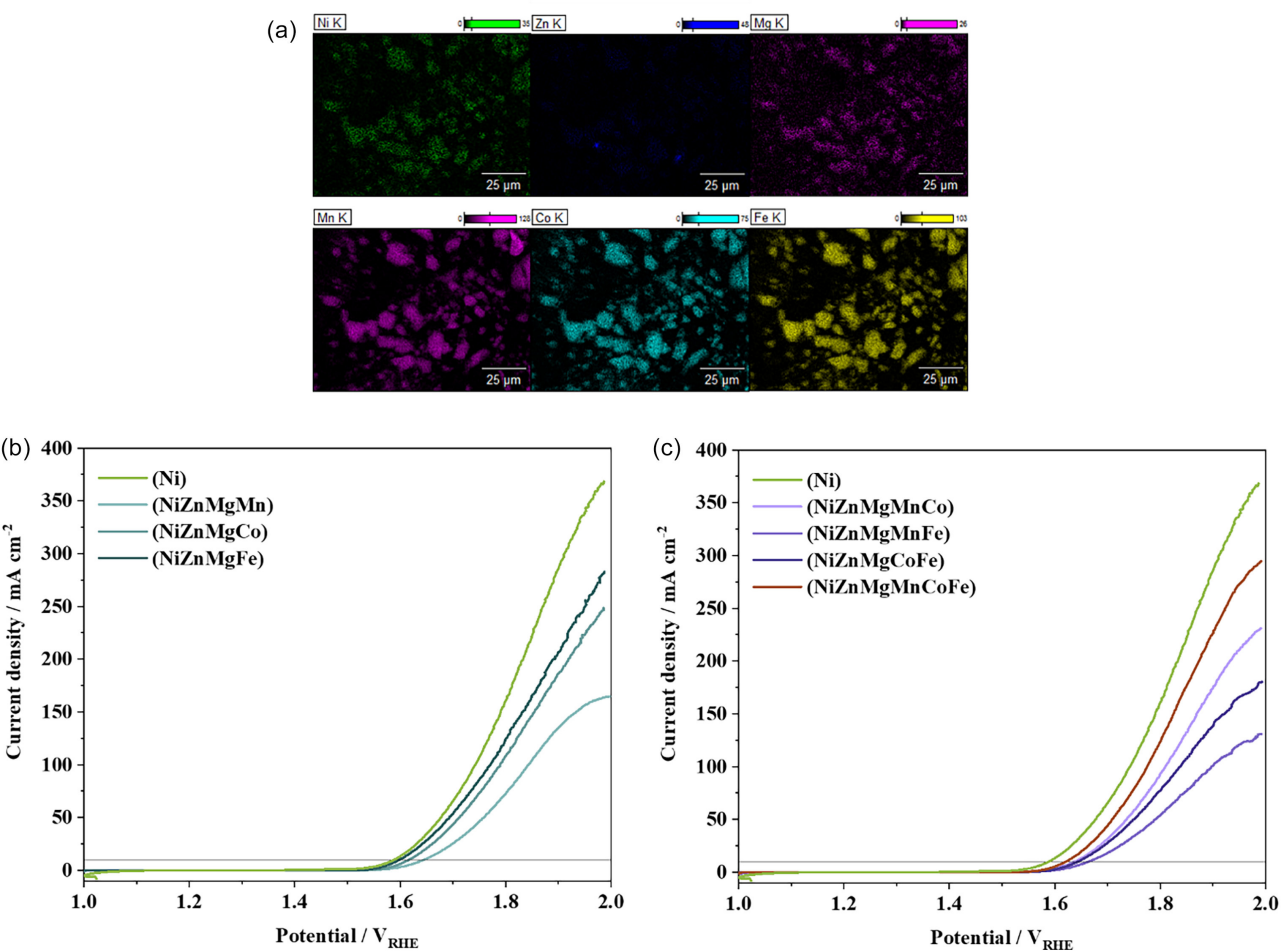
(a) EDX mapping of (NiMgZnMnCoFe)(MnCoFe)_2_O_4_, (b) and (c) LSV measurements of HEOs, performed in 1 M KOH, further impedance, CV scans, and Tafel analysis given in Figure S24, Supporting Information.

The XRD measurements (Figure S22, Supporting Information) show, as with previous materials, NiO in each composition, the crystallite sizes are small due to highly amorphous materials with high lattice distortion (Table S9, Supporting Information), the intensities differ slightly with varying elemental ratios. HR‐TEM images reveal a lattice plane distance of ∼0.23 nm, which is similar to the previous materials (Figure S23, Supporting Information). Complete incorporation of Ni into the crystal structure due to a high entropy stabilization effect is not observed. Investigation of the catalyst that theoretically contains all the listed elements at the A position shows a homogeneous distribution in EDX mapping (Figure 6a) over the entire area, with the measured atomic‐% corresponding to the intended ratios in (NiZnMgMnCoFe)(MnCoFe)_2_O_4_ (6% Ni, 4% Zn, 8% Mg, 28% Mn, 23% Co, 31% Fe). The Ni, Zn, and Mn contents are slightly lower than in (NiZnMg)(MnCoFe)_2_O_4_, as the A position is also occupied by Mn, Co, and Fe. The Raman and DRIFT measurements given in Figures S18 and S19 (Supporting Information) do not differ from the previous ones, with hardly any signals of the spinel structure and signals from organic residues on the surface of the particles. An application of the HEOs in electrocatalysis shows no improvement in overpotential through the introduction of high‐entropy stabilization (Figure 6b,c).

**FIGURE 7:**
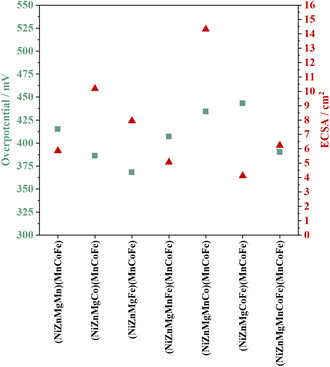
Correlation of overpotential and ECSA in high entropy spinels.

A comparison of all complex catalysts is shown in Figure 7. Increasing the amount of Fe, Co, or Mn in the structure has no enhancing effect compared to the best material of this study, (Ni)(MnCoFe)_2_O_4_. An increase in the Fe content is more beneficial than an increase in the Co content, corresponding to previous results on (Co_*x*_MnFe)_3_O_4_. The addition of a combination of two of these three elements at the A position further reduces the activity in all cases. The highest current density at 2.0 V_RHE_ is achieved when all three elements are added at the A position. Since high entropy stabilization would be achieved with at least five elements in equimolar amounts at one crystallographic position, this could be an indication that high entropy begins to impact activity. It is not certain that all elements are present in exactly equimolar amounts at the A position, especially since three of them are stable in both + 3 and + 2. Furthermore, Ni is incorporated in smaller amounts due to the formation of NiO. Therefore, (NiZnMgMnCoFe)(MnCoFe)_2_O_4_ is the first material in which high entropy stabilization at the A position should be present, even with a partially inverted spinel structure. However, the activity still cannot reach the values achieved by (Ni)(MnCoFe)_2_O_4_. The determination of CTR and ECSA is given in Table 4; the results of these values confirm the trend observed in the overpotential with an increased CTR and decreased ECSA with more A‐cations.

**TABLE 4:** Summary of electrocatalytic results, overpotential, CTR, ECSA (measured in 1 M KOH), and BET surface area for comparison of high‐entropy spinels, (A)(MnCoFe)_2_O_4_.

Composition A position	Overpotential@10 mA, mV	CTR, Ω	ECSA, cm^2^ a	BET, m^2^g^−1^
(NiZnMgMn)	415	6.2	5.86	187
(NiZnMgCo)	386	2.9	10.18	134
(NiZnMgFe)	368	2.5	7.93	170
(NiZnMgMnCo)	407	3.1	14.31	167
(NiZnMgMnFe)	434	4.6	5.06	172
(NiZnMgCoFe)	415	6.2	4.12	175
(NiZnMgMnCoFe)	390	6.7	6.23	173

a Calculated from double layer capacitance, fit given in Figure S25, Supporting Information.

### 3.4 Switch to M_3_O_4_

**FIGURE 8:**
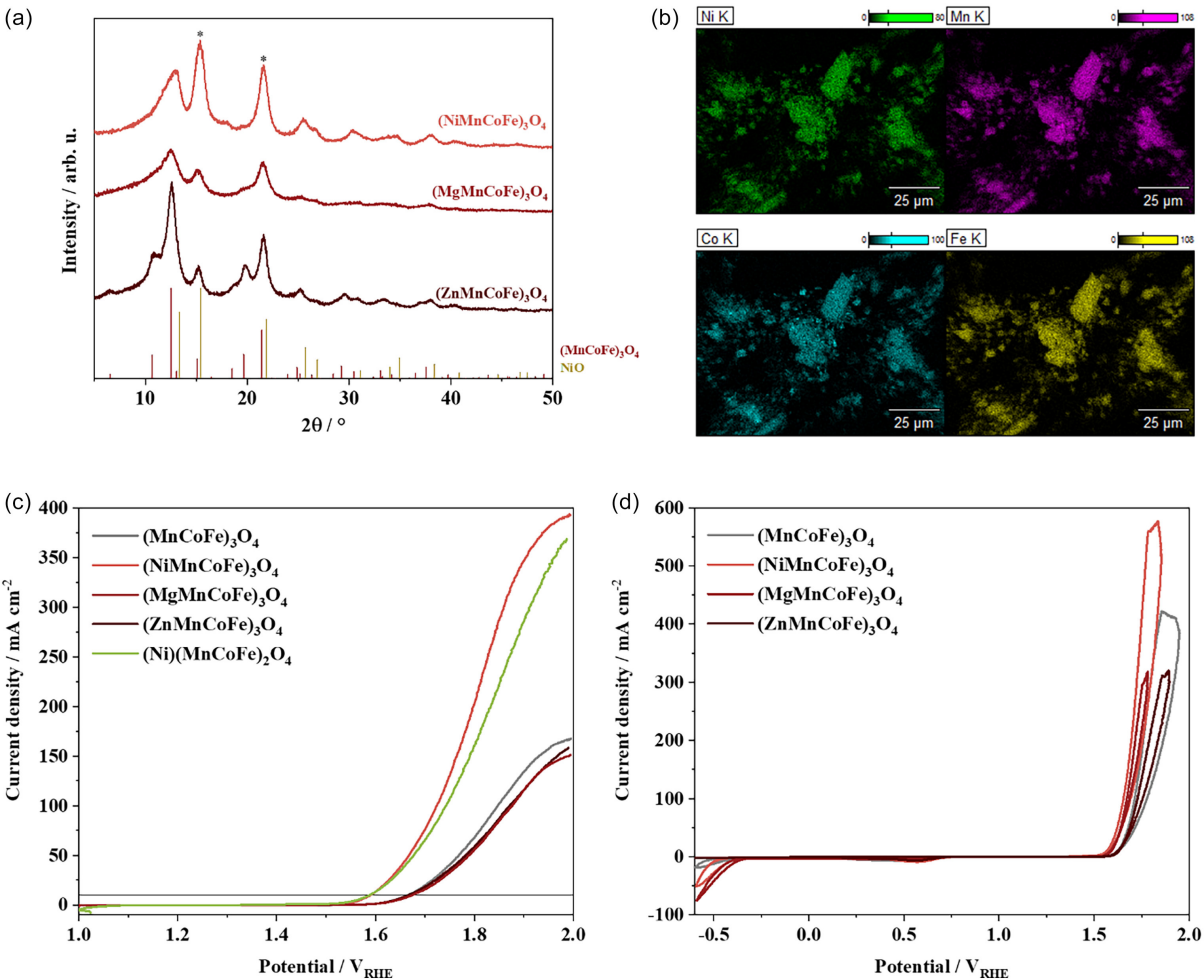
(a) XRD measurements of M_3_O_4_‐type spinels, Raman and DRIFT given in Figure S26, Supporting Information (b) EDX mapping of (NiMnCoFe)_3_O_4_, (c) LSV measurements of M_3_O_4_‐type spinels and (d) corresponding CV scans, performed in 1 M KOH.

In the final step of investigating the effects of structural and compositional changes in spinels, the formula is changed to M_3_O_4_. Theoretical calculations show that only one +2 cation can be incorporated into the structure in equimolar amounts because two cations would prevent charge neutrality due to the 1:2 ratio of +2 to +3 cations. Therefore, Zn, Mg, and Ni are added as single elements for comparison with previous materials. Spinels are formed with each element, although the expected NiO is present again with Ni (Figure 8a). Zn and Mg addition does not result in a side phase, but with Zn, the nanoparticles are more crystalline and larger than with Mg (Table S10, Supporting Information). Compared to their corresponding A(MnCoFe)_2_O_4_ spinel, the crystallite sizes are slightly smaller. The mapping data (Figure 8b) from (NiMnCoFe)_3_O_4_ shows a homogeneous distribution and the detected atomic‐% of ∼26% Mn, 26% Fe, 24% Co, and 25% Ni fit the intended ratios. When comparing the materials in electrocatalysis, only the Ni‐containing spinel can reach the so far best material (Figure 8c). In line with previous results on Ni‐content, there is no significant difference between 25% and 33% of Ni in the catalyst. CV scans confirm the stability over the entire potential window with some activity of (MgMnCoFe)_3_O_4_ towards HER at high overpotentials (Figure 8d). The CTR (Table 5) follows the trend observed in overpotentials, as no influence of a change in stoichiometry is apparent, similar to the previous materials. The ECSA for catalysts with low overpotentials is high, but a high ECSA does not automatically lead to a low overpotential, as can be seen again in both materials with only Mg added.

**TABLE 5:** Summary of electrocatalytic results, overpotential, CTR, ECSA (measured in 1 M KOH), and BET surface area for M_3_O_4_ type spinels, impedance measurements and Tafel analysis given in Figure S27, Supporting Information.

Composition	Overpotential@10 mA, mV	CTR, Ω	ECSA, cm^2^ a	BET, m^2^ g^−1^
(NiMnCoFe)_3_O_4_	358	2.1	9.66	173
(ZnMnCoFe)_3_O_4_	444	8.7	1.95	140
(MgMnCoFe)_3_O_4_	438	6.5	0.62	153

a Calculated from double layer capacitance, fit given in Figure S28, Supporting Information.

This study examined the impact of stabilizing the +3 oxidation state of the redox‐active elements Mn, Co, and Fe by adding various amounts of both +2 redox‐active and ‐inactive elements. This was also paired with introducing the high‐entropy effect to potentially increase activity further. After investigating the possible combinations of elements to add to the base spinel composition, a clear relationship between composition and activity became apparent. Adding Ni led to the formation of a spinel/NiO‐type composite that showed exceptional activity compared to all other compositions, even with low percentages of NiO and the addition of Zn.

In contrast, the high‐entropy effect was not as beneficial as assumed. Changes in material properties, such as conductivity and CTR, overcome stabilization in a higher energetic state. Based on these results, it is likely that the underlying OER mechanism depends on redox‐active elements in a +2 or +3 oxidation state. Thus, the activity of introducing stabilizing elements of other oxidation states will be further studied in the future.

## 4 Conclusion

We prepared multiple spinel nanoparticles of varying compositions by a low‐temperature, microwave‐assisted synthesis. Different redox‐active and ‐inactive metals were introduced into the A position of AB_2_O_4_ spinels to study their stabilizing and synergistic effects by the redox‐active elements Co, Fe, and Mn, as well as on the OER overpotential. The introduction of Ni into the (MnCoFe)_3_O_4_ spinel and the formation of a NiO/spinel composite resulted in a low overpotential of 357 mV, which remained consistent regardless of the amount of Ni used. The activity remained stable with the additional introduction of Zn, while all other elements and combinations thereof decreased the OER activity. This highlights the sensitivity of catalytic systems to compositional changes. This effect could not be reversed, even by introducing a potential high‐entropy stabilization into the system. Thus, we demonstrated the limitations of the composition–activity relationship and showcased the potential for optimization with different element combinations, as opposed to high‐entropy stabilization.

## Conflicts of Interest

The authors declare no conflicts of interest.

## Supporting information

Supplementary Material

## Data Availability

The data that support the findings of this study are available from the corresponding author upon reasonable request.
