# Supplementary material for: Adjusting the Composition of Novel Earth‐Abundant Transition Metal‐Based Oxide Nanoparticles for Electrocatalytic Oxygen Evolution Reaction
Source: ChemSusChem. 2026 Jul 11;19(14):e70880. doi: 10.1002/cssc.70880 (PMC13354971; doi:10.1002/cssc.70880)
Supplement: Supplementary file 1 — Supplementary Material [file CSSC-19-e70880-s001.pdf]

Supporting Information

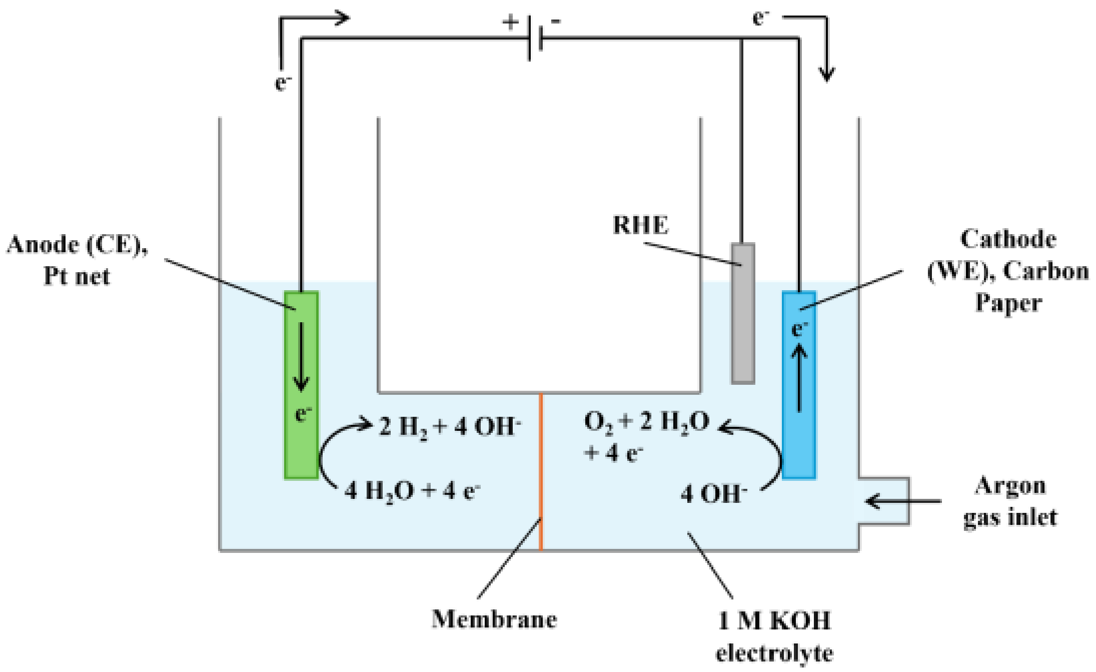

Figure S1. Schematic electrochemical setup of the H-cell.

Table S1. Producer and purity of all used chemicals.

Chemical	Producer	Purity / %
$Ni(acac)_2$	Sigma Aldrich	97
$Zn(acac)_2$	TCI	≥96
$Mg(acac)_2$	TCI	≥98
$Mn(acac)_3$	TCI	≥98
$Co(acac)_3$	abcr	98
$Fe(acac)_3$	Acros Organics	≥99
1-Phenylethanol	Sigma Aldrich	98
2-Propanol	VWR	99.5
5wt% Nafion	Alfa Aesar	/
KOH	Fisher sientific	≥99

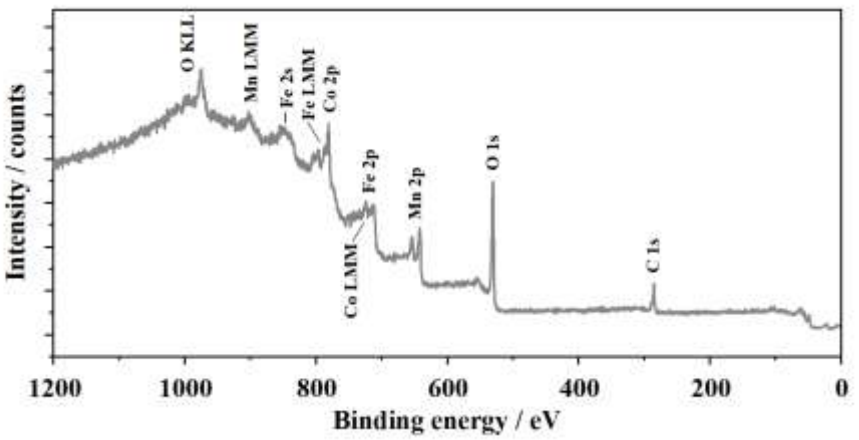

Figure S2. XPS Survey scan of $(MnCoFe)_3O_4$ with assigned signals.

Table S2. Position and calculated at% of elements contained.

Signal	Position / eV	At-%
Mn 2p3/2	641.6	7.1
Fe 2p	711.2	8.0
Co 2p3/2	780.4	9.6
O 1s	530.0	50.8
C 1s	284.8	24.6

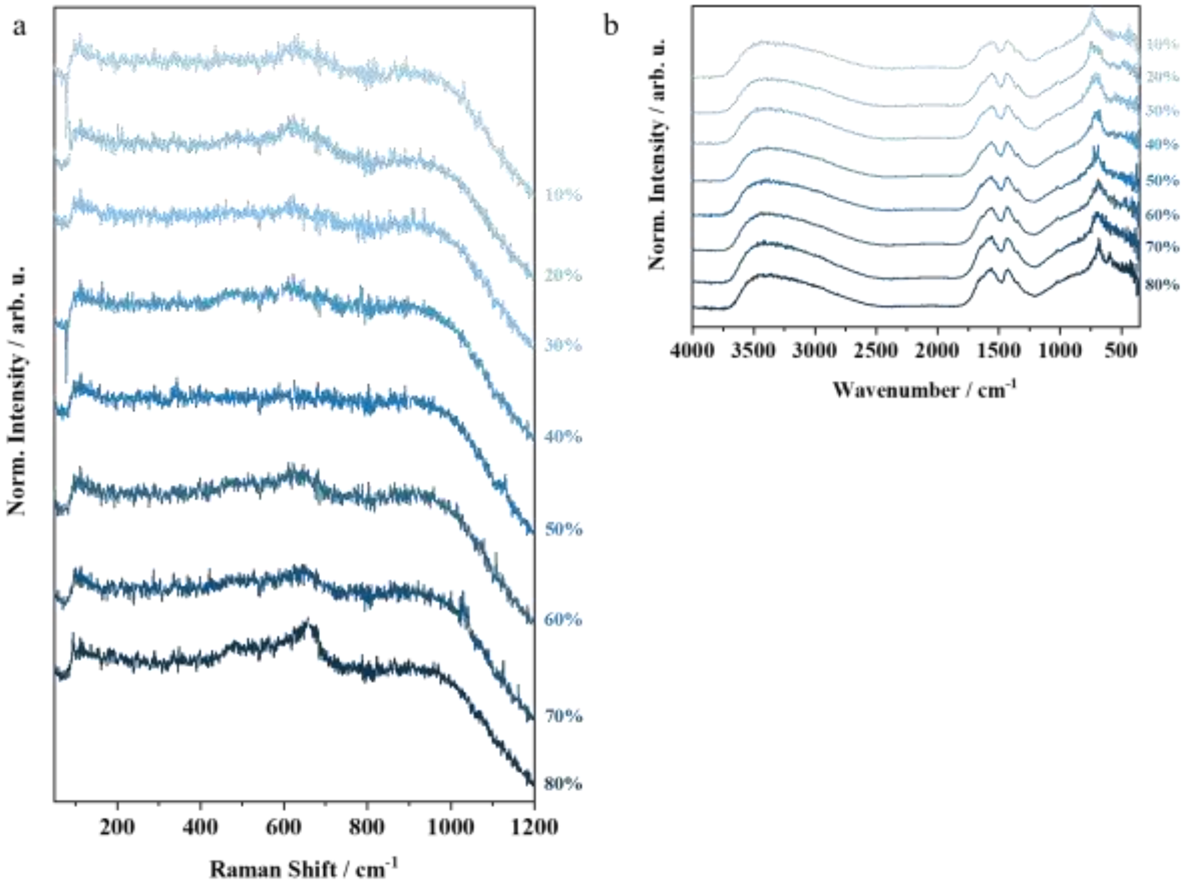

Figure S3. Raman (a) and DRIFT (b) measurements of $(Co_xMnFe)_3O_4$.

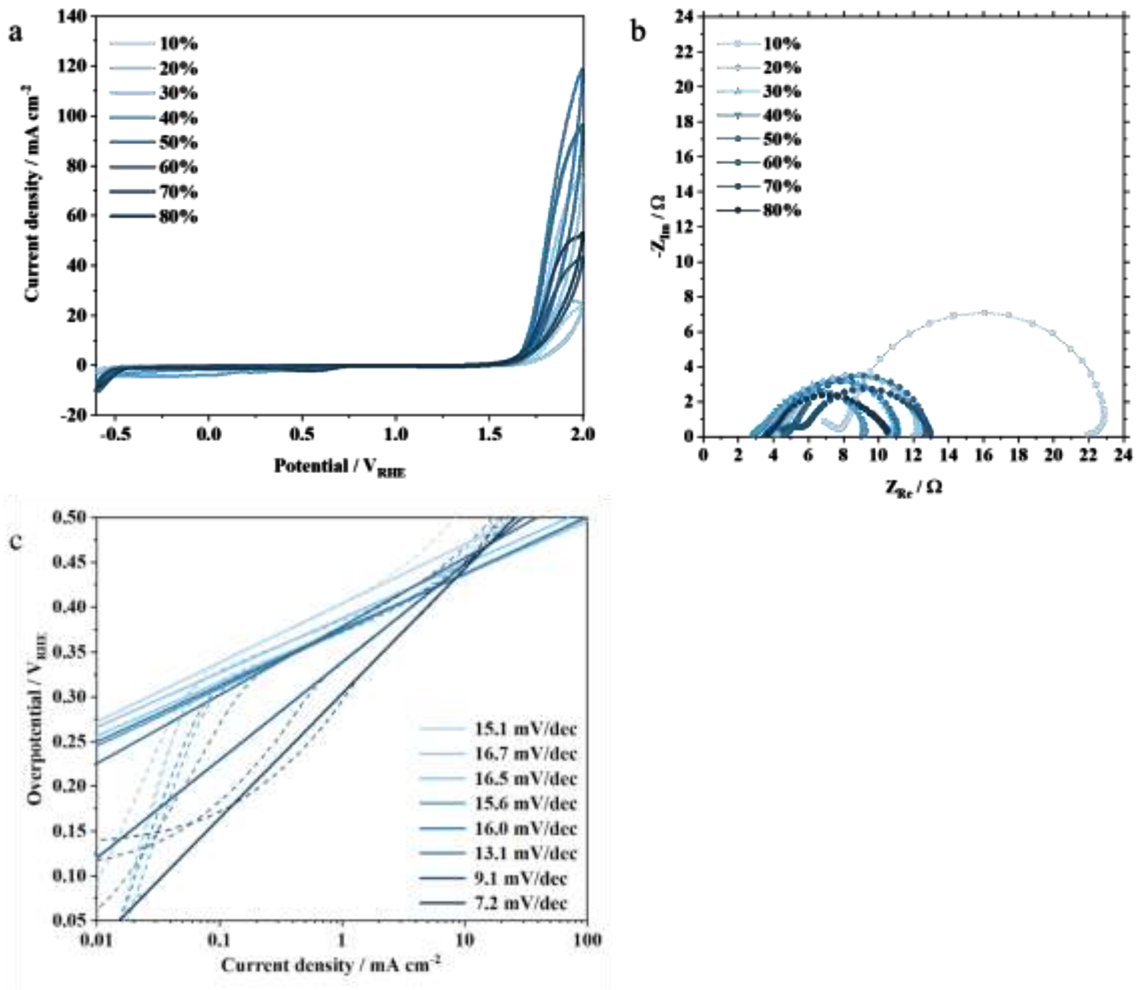

Figure S4. CV scans (a), impedance for CTR determination (b) and Tafel analysis (c) of $(Co_xMnFe)_3O_4$.

Table S3. Summary of electrocatalytic values and surface area.

Co-amount / %	Overpotential@10mA / mV	CTR / Ω	ECSA / cm^2	BET / m^2/g	Crystallite size / nm
10	514	15.2	3.81	170	11.7
20	457	8.2	2.51	169	11.5
30	463	7.3	1.96	163	12.2
40	447	6.1	4.39	172	11.1
50	450	7.1	3.98	165	8.2
60	467	7.8	5.74	158	5.8
70	450	7.4	5.66	141	5.8
80	437	6.9	5.30	126	6.7

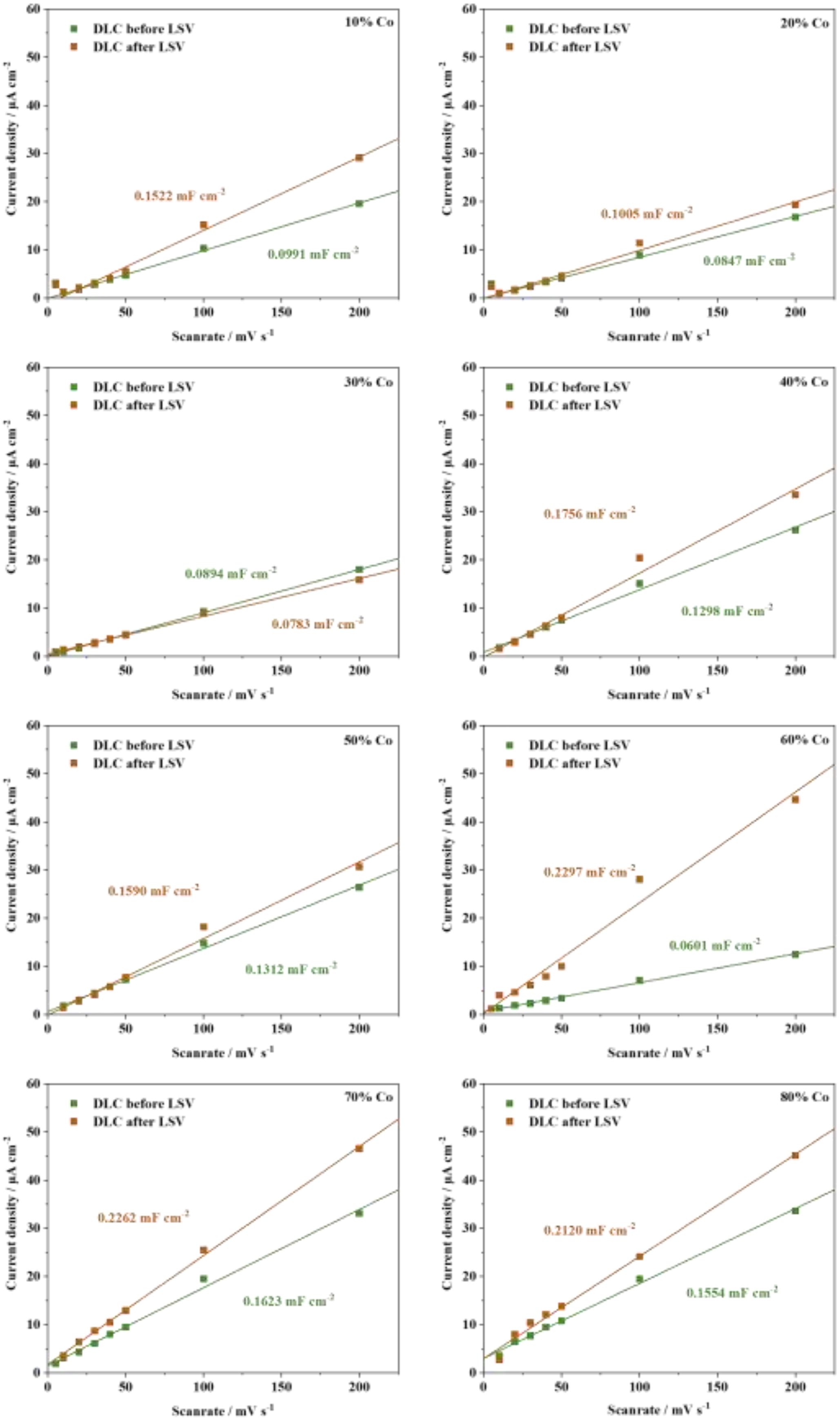

Figure S5. DLC plots before and after LSV for ECSA determination of different Co-amounts in $(Co_xMnFe)_3O_4$.

Table S4. Crystallite sizes, calculated with Scherrer equation, of $A(MnCoFe)_2O_4$.

Composition	Crystallite size / nm
$(MnCoFe)_3O_4$	2.6
$(Ni)(MnCoFe)_2O_4$	3.3
$(Mg)(MnCoFe)_2O_4$	6.2
$(Zn)(MnCoFe)_2O_4$	10.7

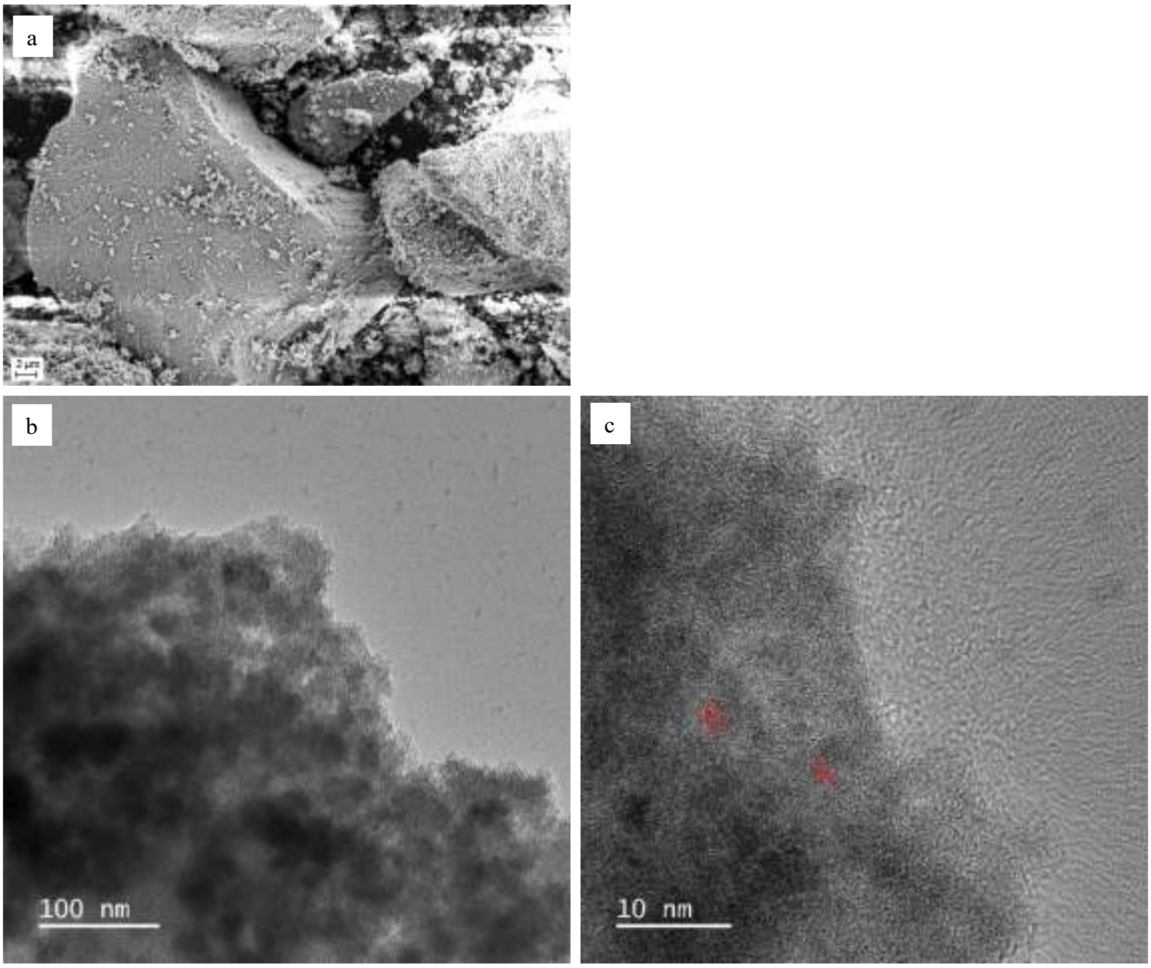

Figure S6. SEM (a) and HR-TEM (b,c) images of $(Ni)(MnCoFe)_2O_4$.

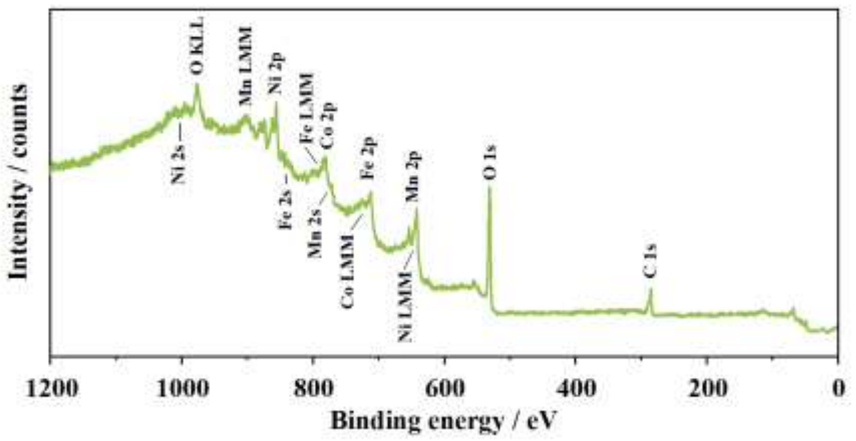

Figure S7. XPS Survey scan of (Ni)$(MnCoFe)_2O_4$ with assigned signals.

Table S5. Position and calculated at% of elements contained.

Signal	Position / eV	At-%
Ni 2p3/2	855.6	9.5
Mn 2p3/2	642.0	8.7
Fe 2p	711.6	7.7
Co 2p3/2	780.4	7.0
O 1s	530.8	43.9
C 1s	284.8	23.2

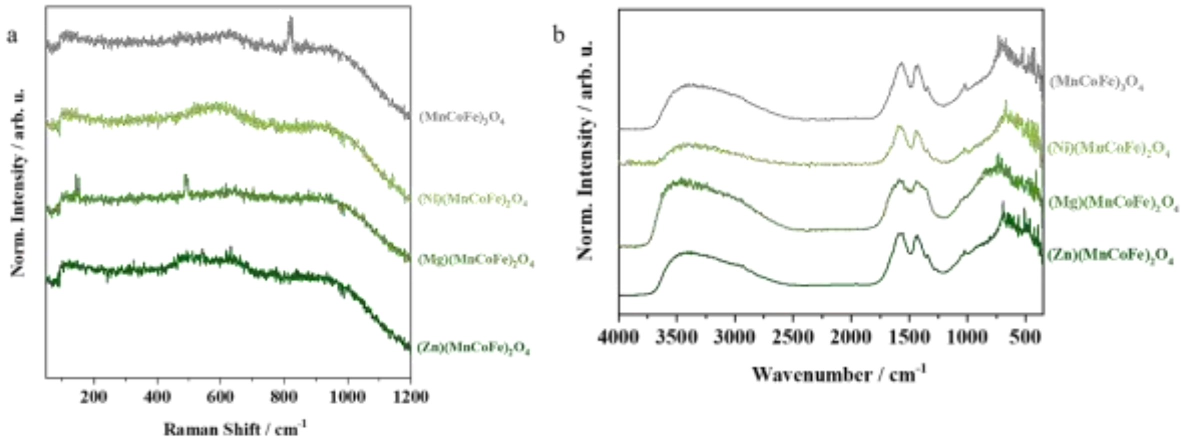

Figure S8. Raman (a) and DRIFT (b) measurements of (A)$(MnCoFe)_2O_4$.

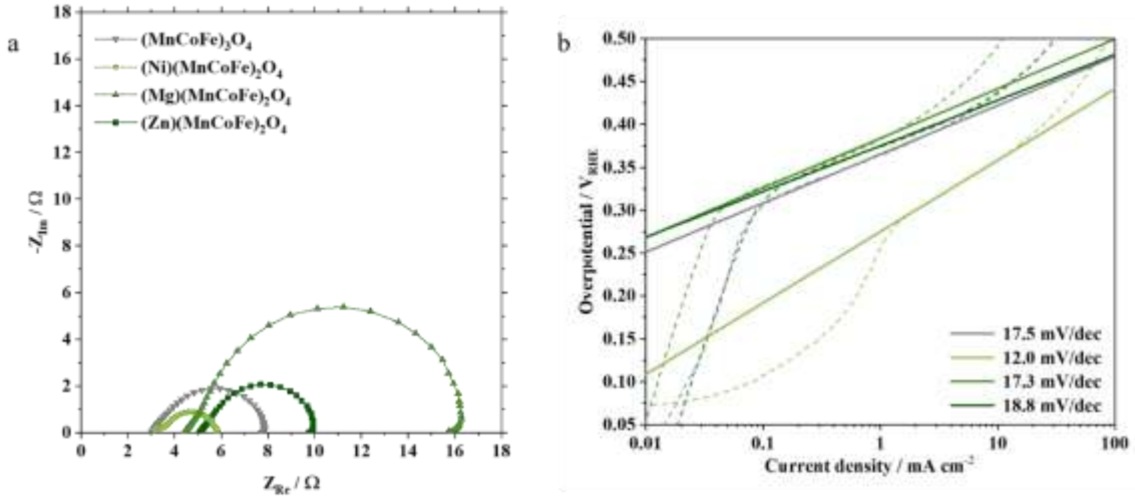

Figure S9. Impedance measurements (a) and Tafel analysis (b) of (A)$(MnCoFe)_2O_4$.

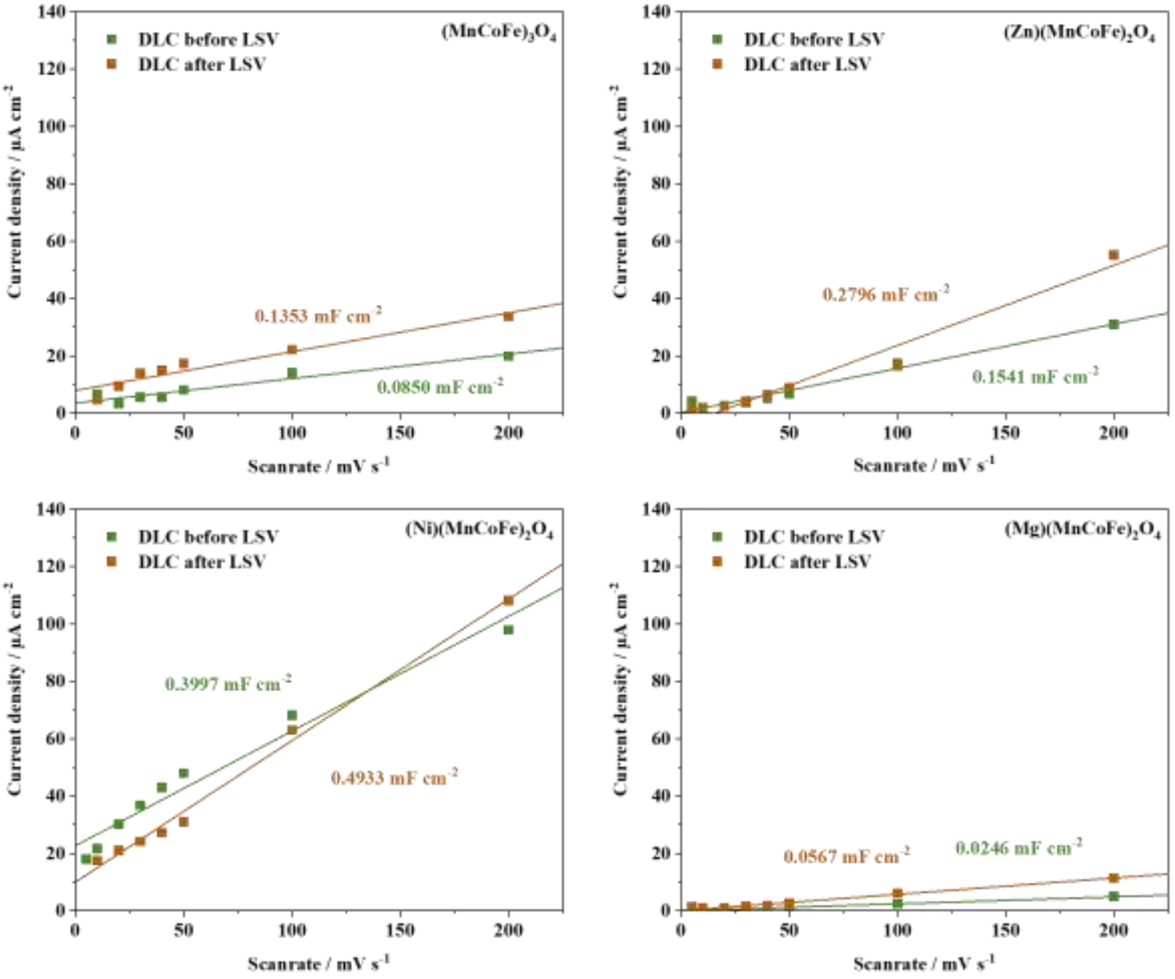

Figure S10. DLC plots before and after LSV for ECSA determination of (A)(MnCoFe)$_2$O$_4$.

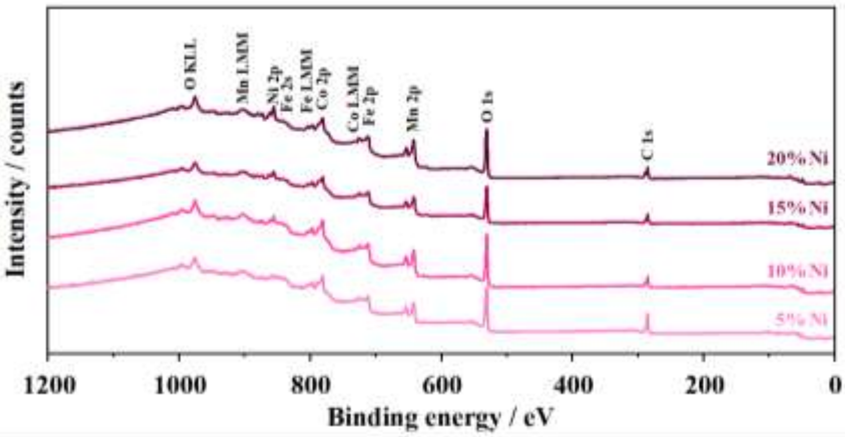

Figure S11. Full XPS Survey scans of $(Ni)_x(MnCoFe)_{3-x}O_4$ with assigned signals.

Table S6a. Signals used for 20% Ni.

Signal	Position / eV	At-%
Ni 2p3/2	854.9	5.2
Mn 2p1/2	653.3	5.1
Fe 2p	711.3	6.4
Co 2p3/2	780.5	7.2
O 1s	530.1	48.9
C 1s	284.8	27.1

Table S6b. Signals used for 15% Ni.

Signal	Position / eV	At-%
Ni 2p3/2	855.2	3.6
Mn 2p	641.2	8.2
Fe 2p	710.4	6.8
Co 2p3/2	780.4	8.2
O 1s	530.0	46.4
C 1s	284.8	26.8

Table S6c. Signals used for 10% Ni.

Signal	Position / eV	At-%
Ni 2p3/2	855.6	2.6
Mn 2p	641.6	8.6
Fe 2p	710.8	7.4
Co 2p3/2	780.4	8.8
O 1s	530.0	48.5
C 1s	284.8	24.2

Table S6d. Signals used for 5% Ni.

Signal	Position / eV	At-%
Ni 2p3/2	856.4	1.29
Mn 2p	641.6	6.3
Fe 2p	710.0	5.1
Co 2p	780.8	7.1
O 1s	530.4	42.9
C 1s	284.8	37.3

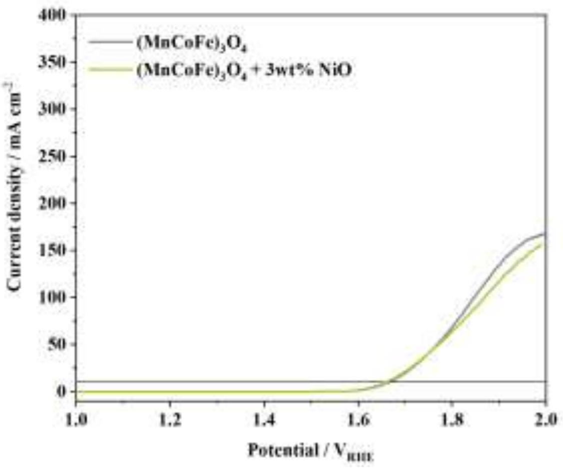

Figure S12. LSV measurement of $(MnCoFe)_3O_4$ with and without post-synthesis added NiO, performed in 1 M KOH.

The synthesis of NiO has been performed with the same parameters as the general spinel synthesis. To avoid microwave vial rupture due to pressure increase, the reaction time has been reduced to 15 min.

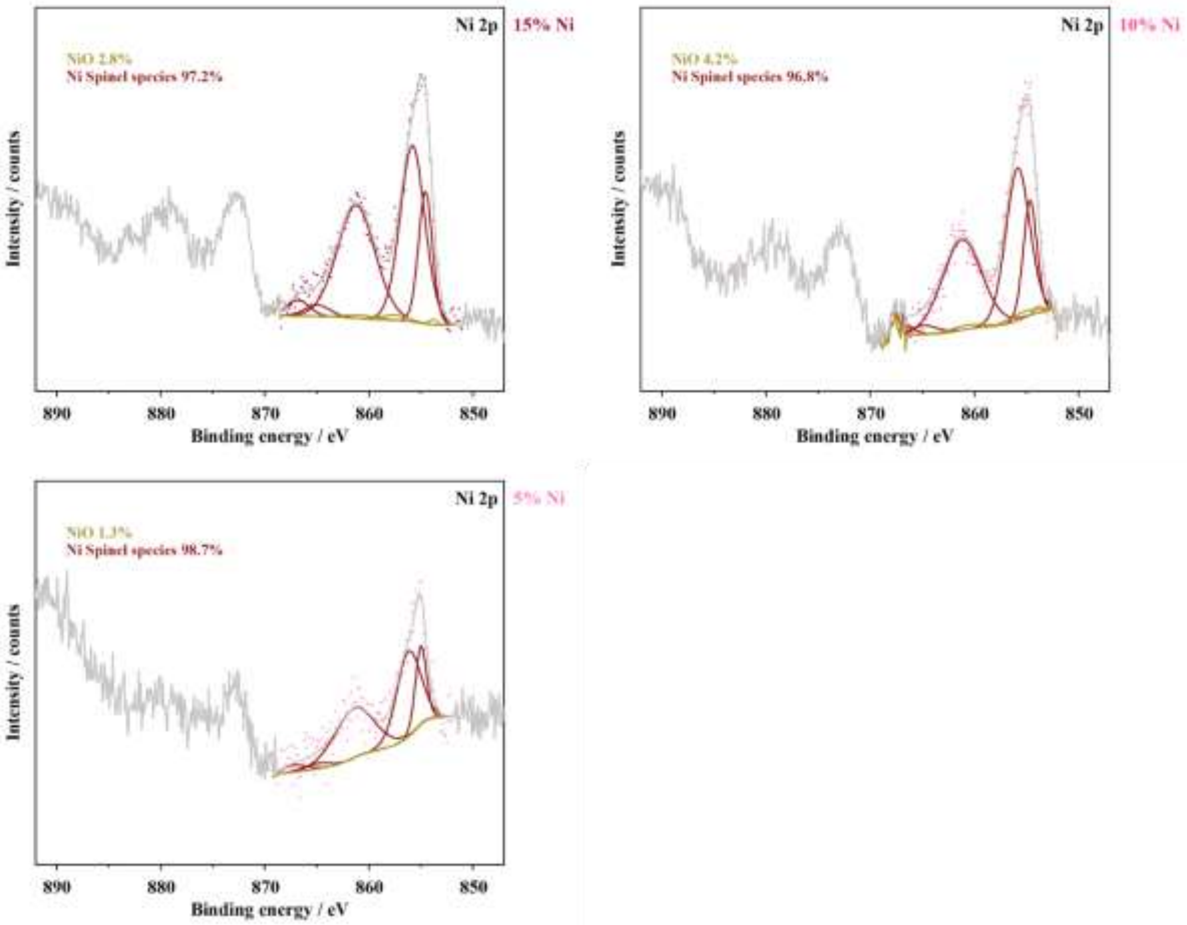

Figure S13. High resolution XPS spectra of 15%, 10% and 5% Ni in $(Ni)_x(MnCoFe)_{3-x}O_4$.

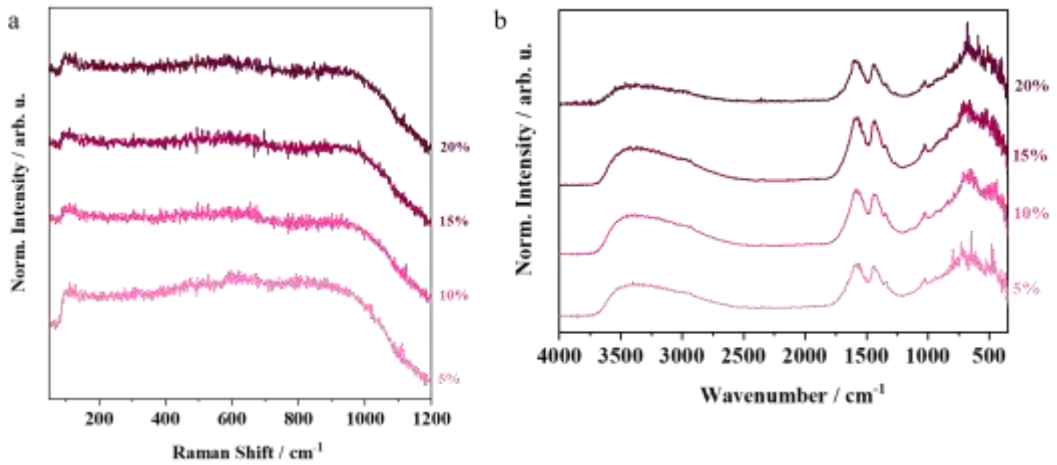

Figure S14. Raman spectra (a) and DRIFT (b) of $(Ni)_x(MnCoFe)_{3-x}O_4$.

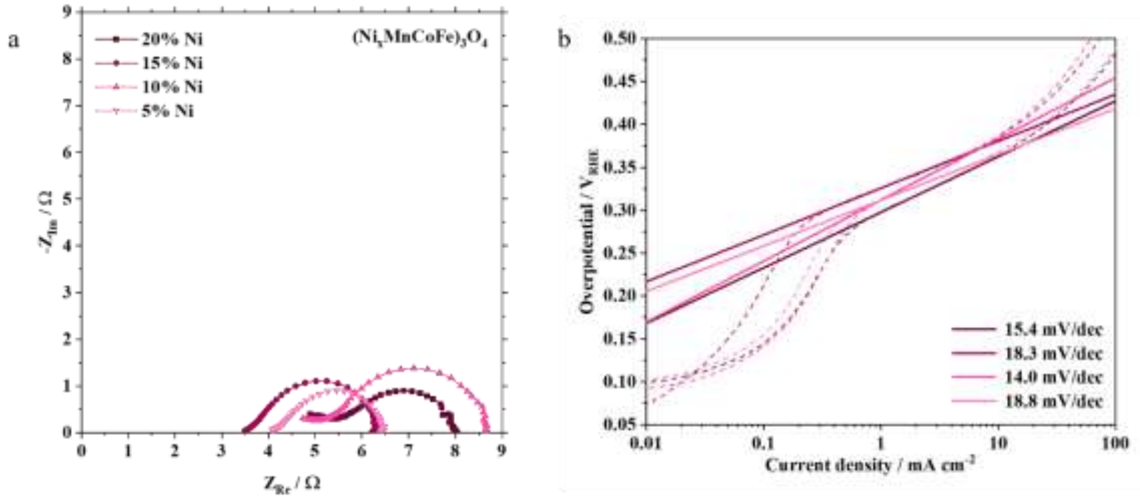

Figure S15. Impedance measurements (a) and Tafel analysis (b) of $(Ni)_x(MnCoFe)_{3-x}O_4$.

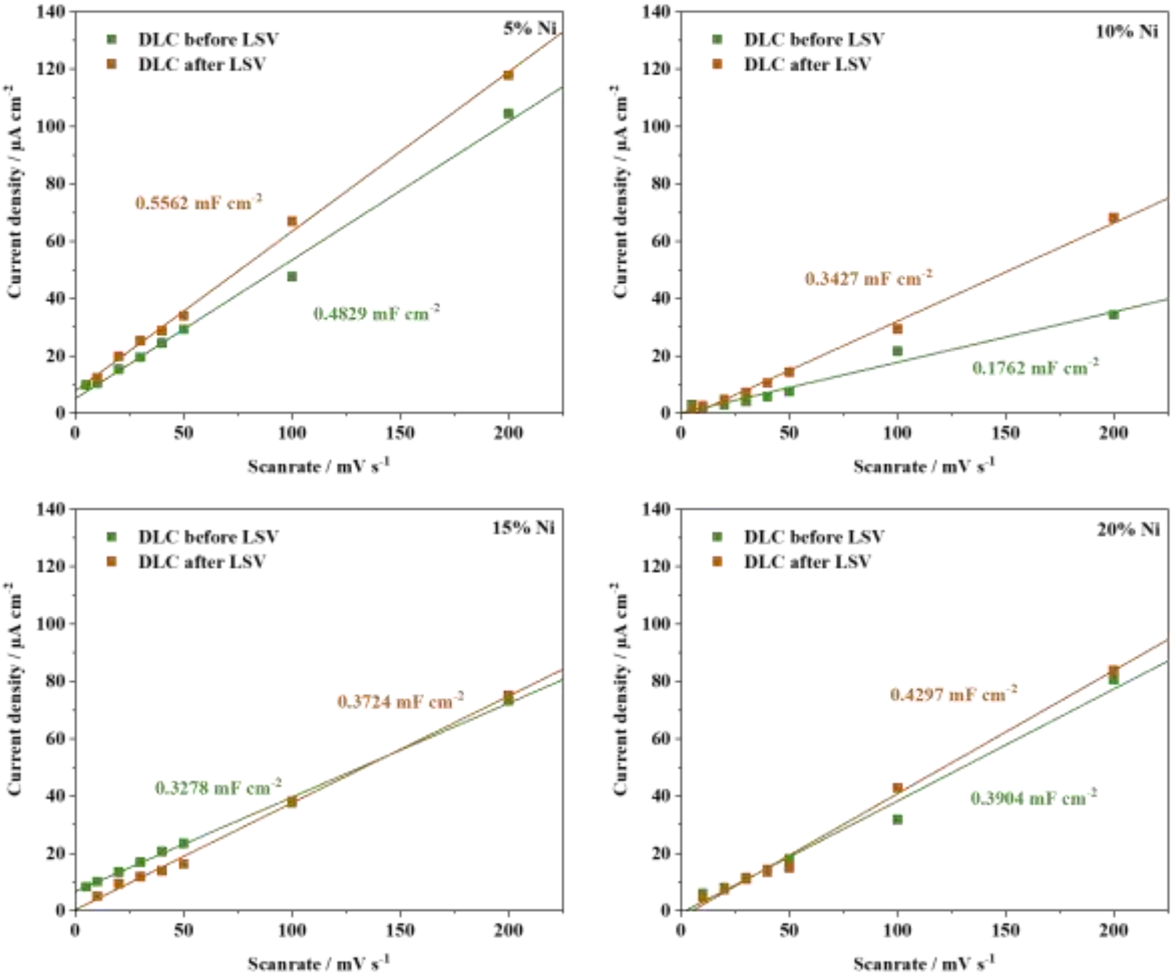

Figure S16. DLC plots before and after LSV for ECSA determination of $(Ni)_x(MnCoFe)_{3-x}O_4$.

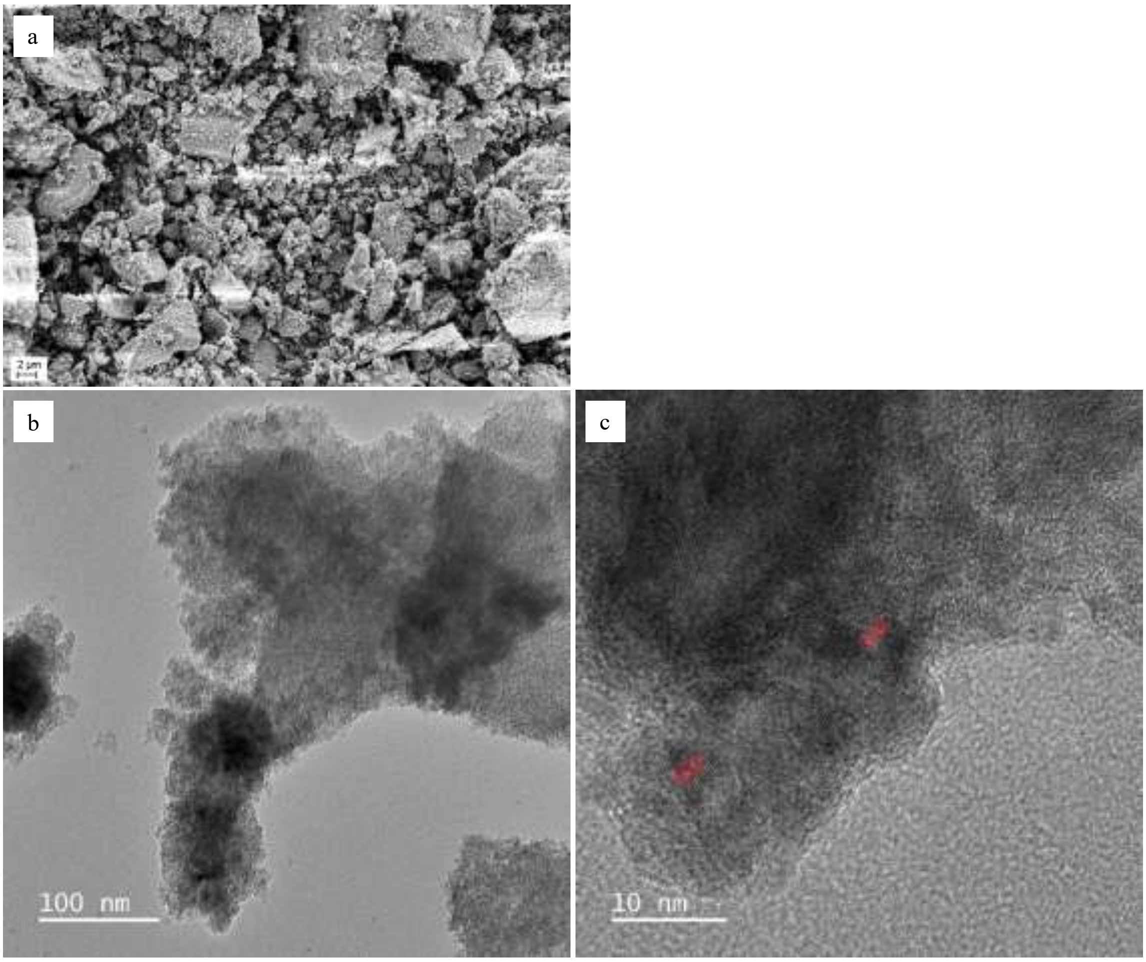

Figure S17. SEM image (a) and Tem images (b,c) of $(NiZn)(MnCoFe)_2O_4$.

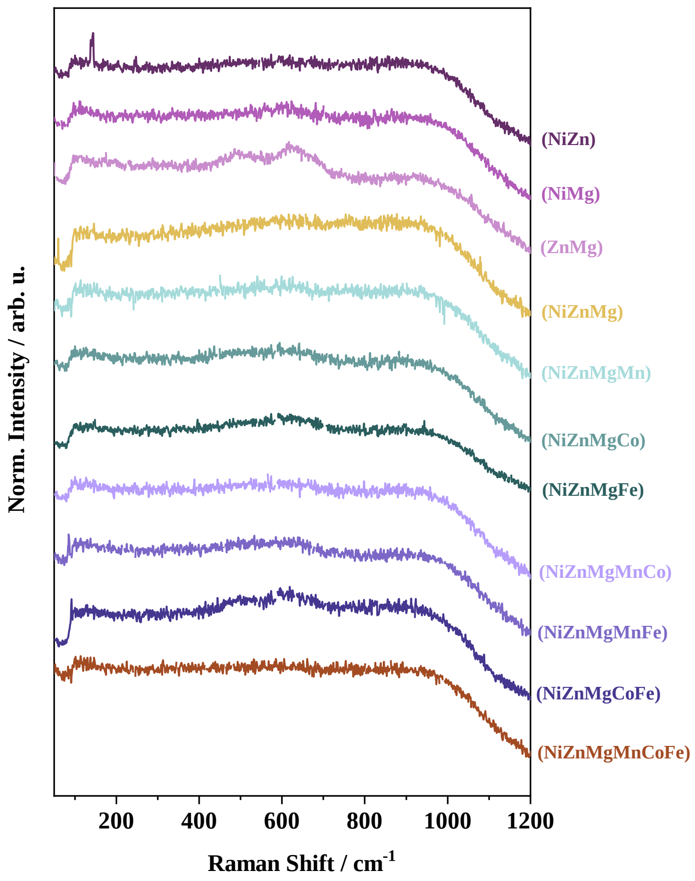

Figure S18. Raman spectra of medium and high entropy spinels with the formula (A)$(MnCoFe)_2O_4$.

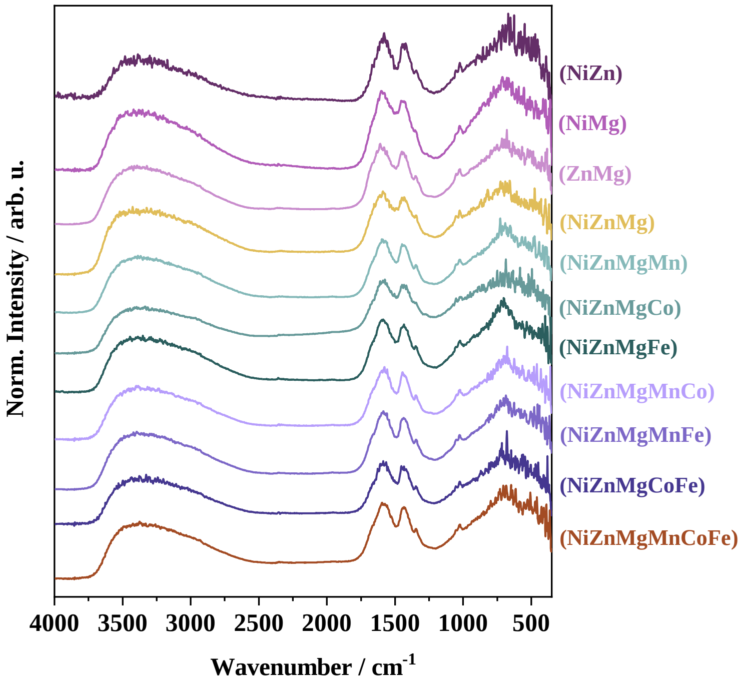

Figure S19. DRIFT measurements of medium and high entropy spinels with the formula (A)$(MnCoFe)_2O_4$.

Table S7. Crystallite sizes, calculated with Scherrer equation, of medium entropy spinels.

Composition	Crystallite size / nm
$(NiZn)(MnCoFe)_2O_4$	3.5
$(NiMg)(MnCoFe)_2O_4$	3.7
$(MgZn)(MnCoFe)_2O_4$	6.5
$(NiZnMg)(MnCoFe)_2O_4$	3.5

Table S8. Position and calculated at% of all elements contained.

Signal	Position / eV	At-%
Ni 2p3/2	854.8	2.8
Mn 2p	641.6	5.5
Fe 2p	711.2	5.8
Co 2p3/2	780.8	7.0
O 1s	530.8	46.8
C 1s	284.8	24.1

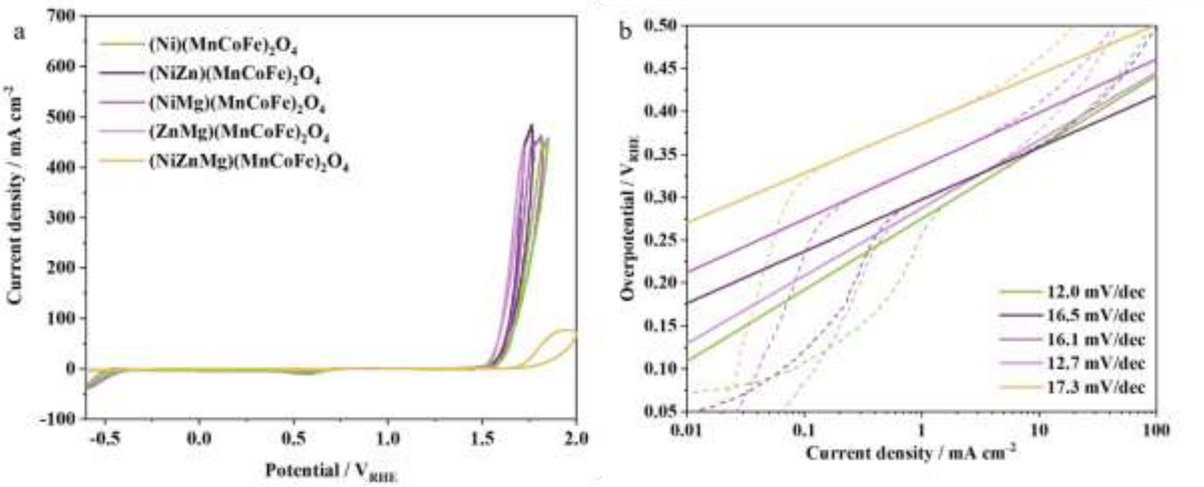

Figure S20. CV scans (a) and Tafel analysis (b) of medium entropy spinels, performed in 1 M KOH.

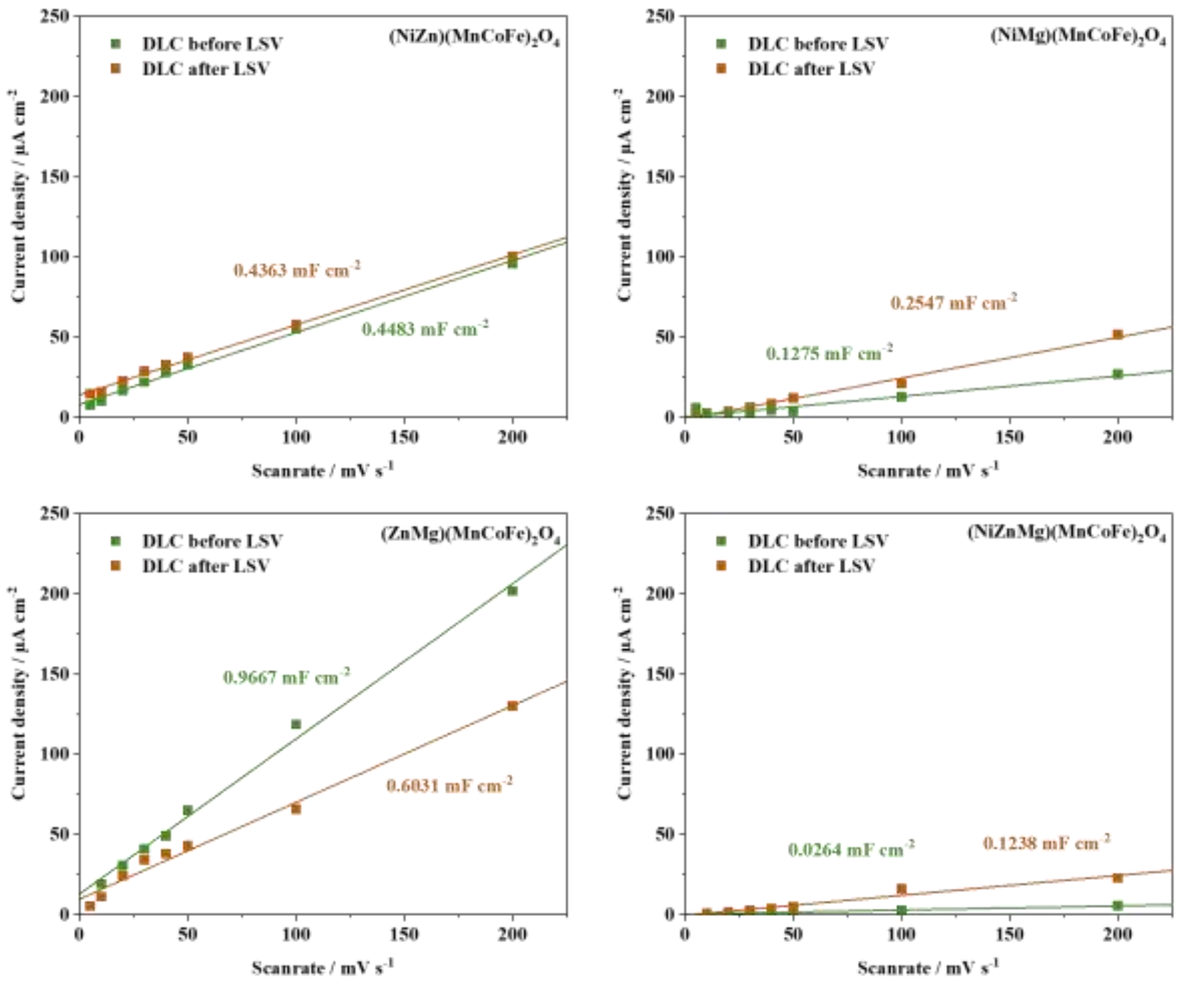

Figure S21. DLC plots before and after LSV for ECSA determination of medium entropy spinels.

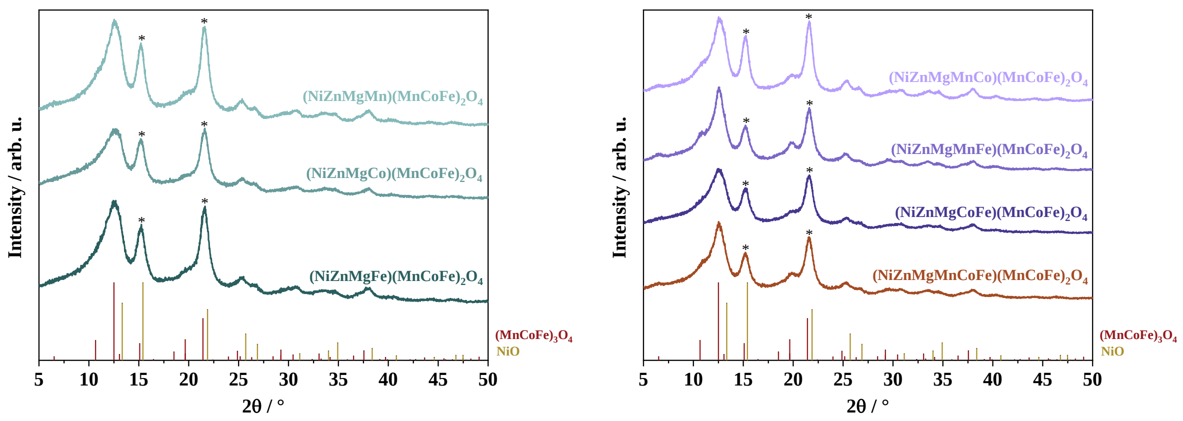

Figure S22. Ag-XRDs of high entropy spinels.

Table S9. Crystallite sizes, calculated with Scherrer equation, of high entropy spinels.

Composition A-Position	Crystallite size / nm
(NiZnMgMn)	4.6
(NiZnMgCo)	4.0
(NiZnMgFe)	4.0
(NiZnMgMnCo)	5.0
(NiZnMgMnFe)	5.8
(NiZnMgCoFe)	4.0
(NiZnMgMnCoFe)	4.9

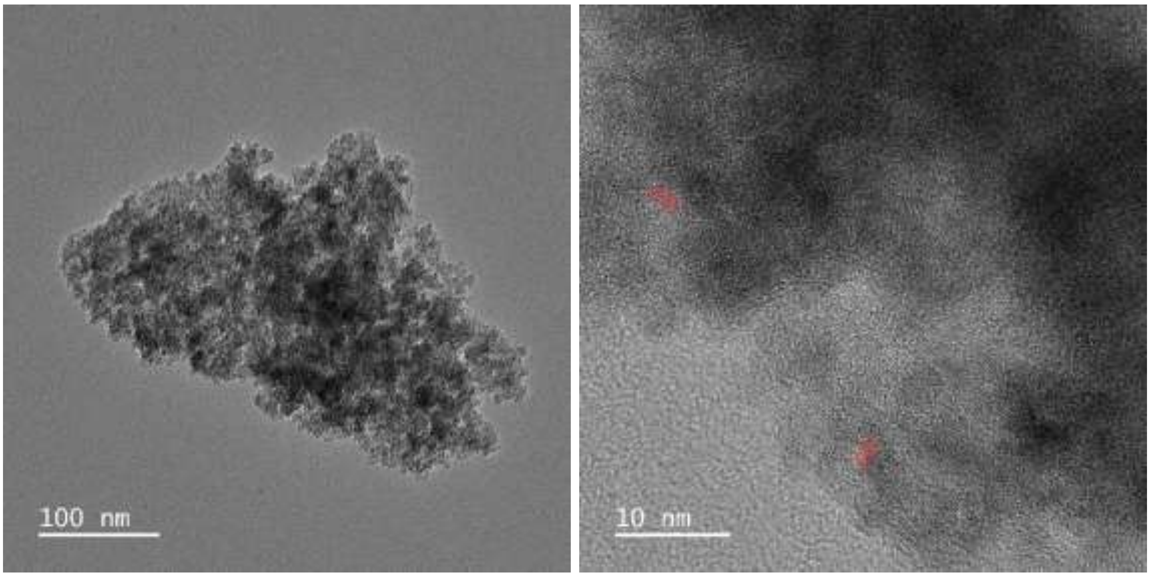

Figure S23: HR-TEM images of (NiZnMgMnCoFe)$(MnCoFe)_2O_4$

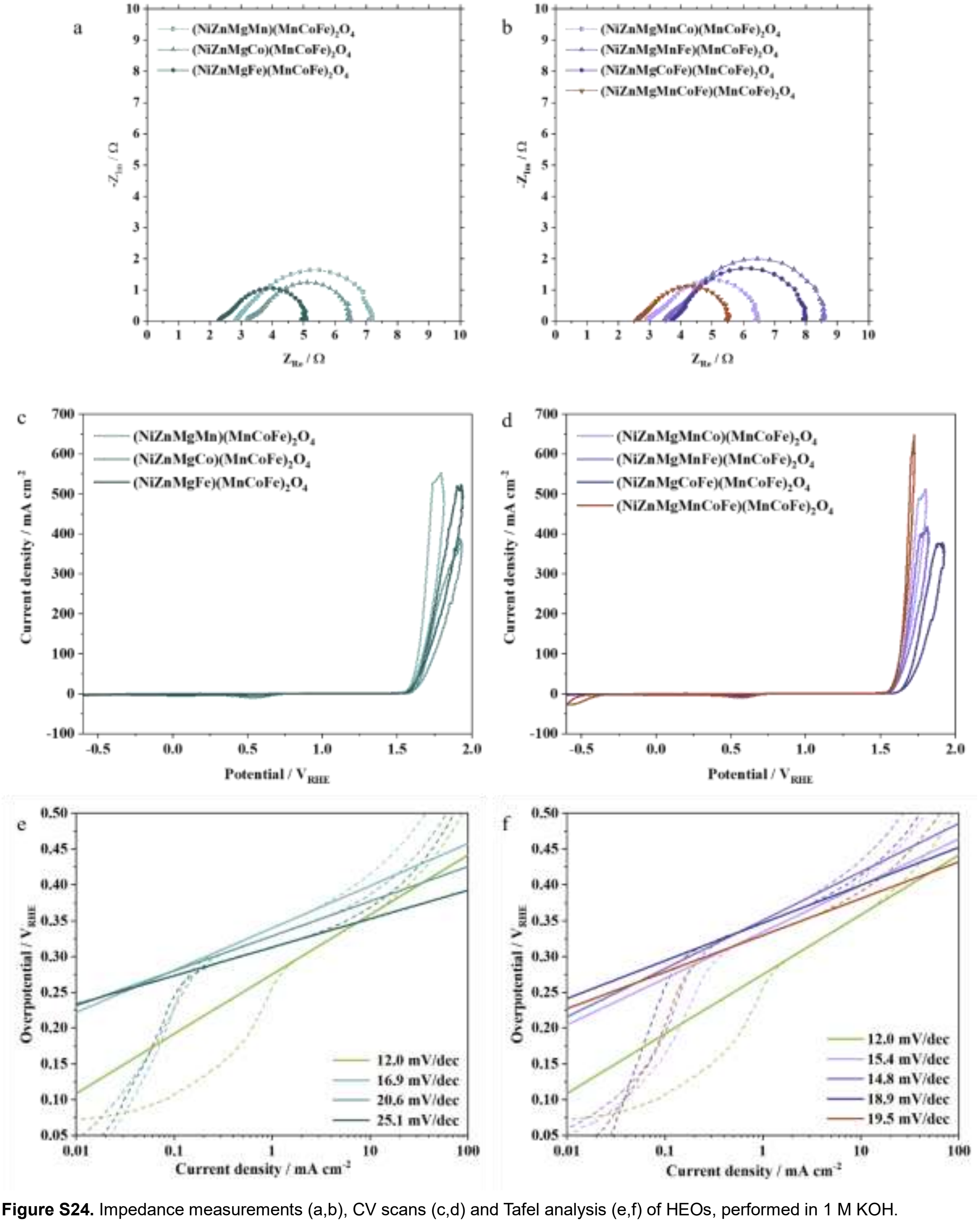

Figure S24. Impedance measurements (a,b), CV scans (c,d) and Tafel analysis (e,f) of HEOs, performed in 1 M KOH.

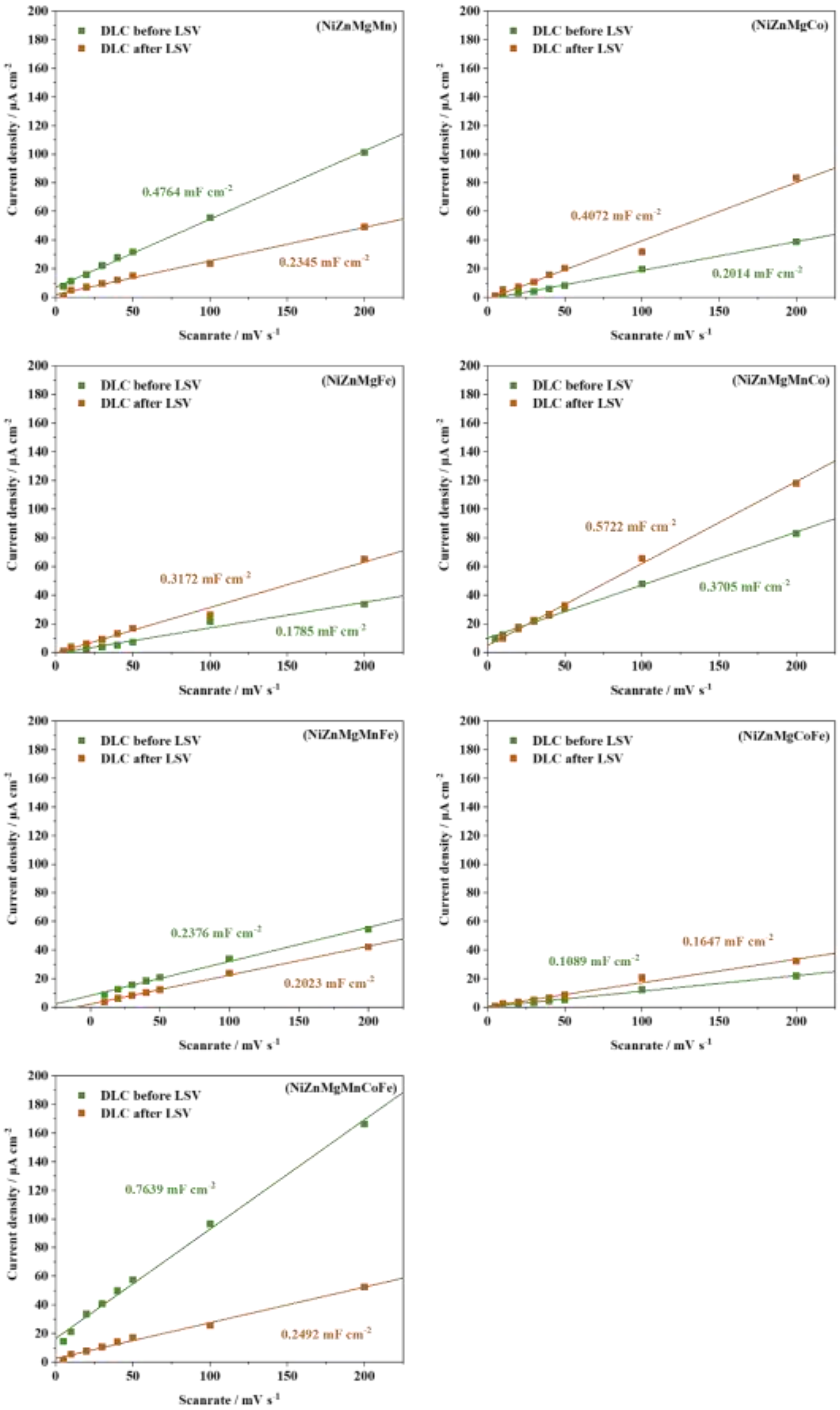

Figure S25. DLC plots before and after LSV for ECSA determination of high entropy spinels.

Table S10. Crystallite sizes, calculated with Scherrer equation, of M_3O_4 type spinels.

Composition	Crystallite size / nm
$(NiMnCoFe)_3O_4$	3.0
$(ZnMnCoFe)_3O_4$	6.5
$(MgMnCoFe)_3O_4$	3.5

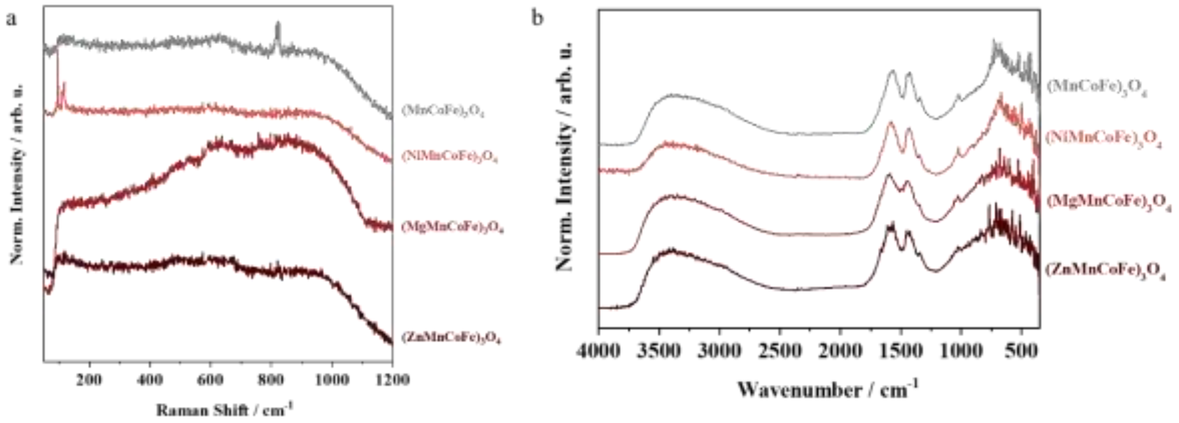

Figure S26. Raman (a) and DRIFT (b) measurements of M_3O_4 type spinels.

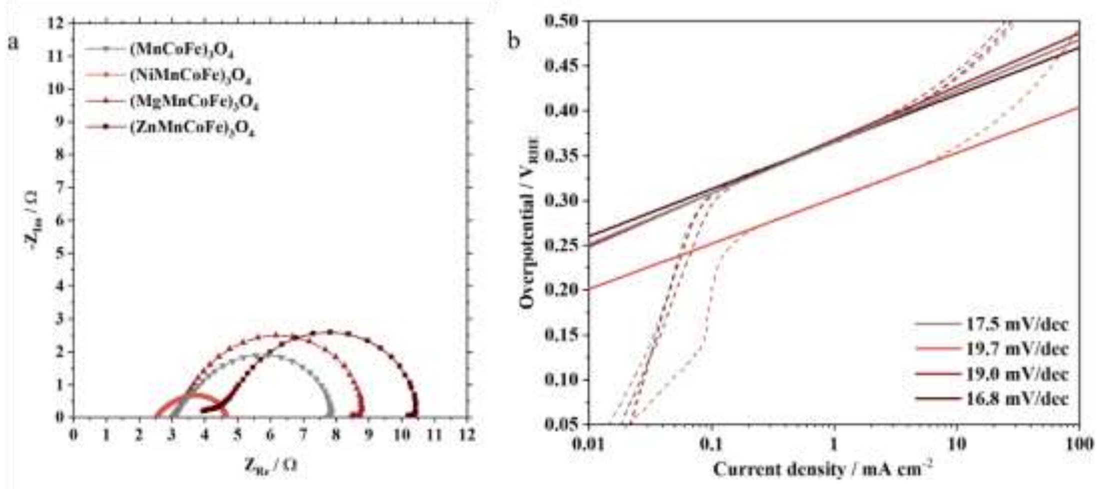

Figure S27. Impedance measurements (a) and Tafel analysis (b) of M_3O_4 type spinels.

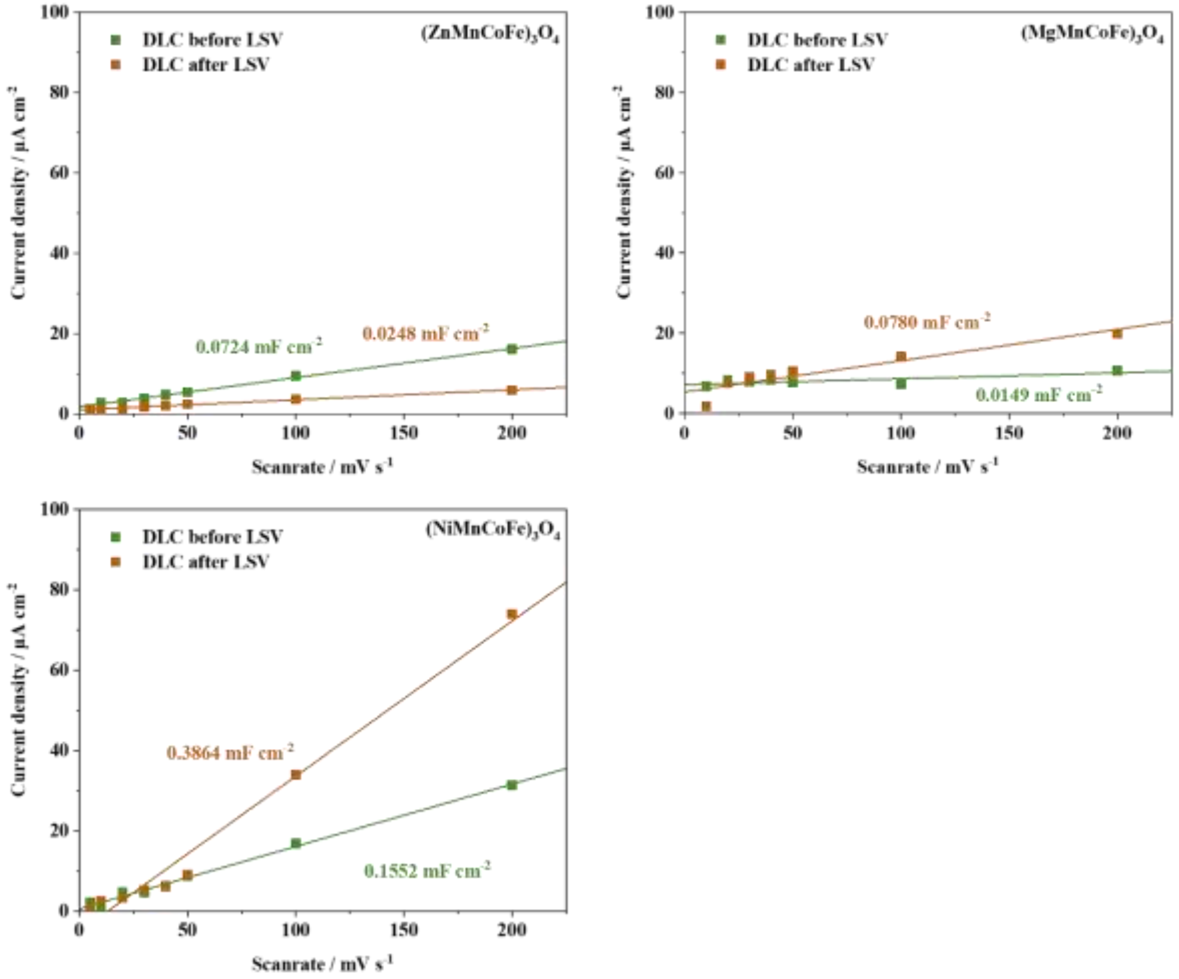

Figure S28. DLC plots before and after LSV for ECSA determination of M_3O_4 type spinels.
